# Countdown to 2015 country case studies: what can analysis of national health financing contribute to understanding MDG 4 and 5 progress?

**DOI:** 10.1186/s12889-016-3403-4

**Published:** 2016-09-12

**Authors:** Carlyn Mann, Courtney Ng, Nadia Akseer, Zulfiqar A Bhutta, Josephine Borghi, Tim Colbourn, Patricia Hernández-Peña, Luis Huicho, Muhammad Ashar Malik, Melisa Martinez-Alvarez, Spy Munthali, Ahmad Shah Salehi, Mekonnen Tadesse, Mohammed Yassin, Peter Berman, On behalf of the Countdown to 2015 Health Finance Working Group, Peter Berman, Josephine Borghi, Ravi Rannan-Eliya, Lara Brearley, Howard Friedman, Nirmala Ravishankar, Rafael Cortes, Gemini Mtei

**Affiliations:** 1 Global Health and Population Department, Harvard T.H. Chan School of Public Health, Boston, MA 02115 USA; 2 Centre for Global Child Health, The Hospital for Sick Children, Toronto, M5G 1X8 Canada; 3 Centre of Excellence in Women and Child Health, Aga Khan University, Karachi, 74800 Pakistan; 4 Centre for Global Child Health, The Hospital for Sick Children, 686 Bay Street, Toronto, Ontario M5G 0A4 Canada; 5 Dalla Lana School of Public Health, University of Toronto, Toronto, Canada; 6 London School of Hygiene & Tropical Medicine, London, WC1E 7HT UK; 7 Institute for Global Health, University College London, London, WC1N 1EH UK; 8 Netherlands Interdisciplinary Demographic Institute (NIDI), The Hague, NL-2511 CV The Netherlands; 9 Centro de Investigación para el Desarrollo Integral y Sostenible and School of Medicine, Universidad Peruana Cayetano Heredia, Lima, Peru; 10 School of Medicine, Universidad Nacional Mayor de San Marcos, Lima, Peru; 11 Instituto Nacional de Salud del Niño, Lima, Peru; 12 Economics Department, University of Malawi, Zomba, Malawi; 13 Ethiopian Public Health Institute, Health System Research, Addis Ababa, Ethiopia

**Keywords:** Health finance, Reproductive health, Newborn health, Maternal health, Child health, Afghanistan, Ethiopia, Malawi, Pakistan, Peru, Tanzania

## Abstract

**Background:**

Countdown to 2015 (Countdown) supported countries to produce case studies that examine how and why progress was made toward the Millennium Development Goals (MDGs) 4 and 5. Analysing how health-financing data explains improvements in RMNCH outcomes was one of the components to the case studies.

**Methods:**

This paper presents a descriptive analysis on health financing from six Countdown case studies (Afghanistan, Ethiopia, Malawi, Pakistan, Peru, and Tanzania), supplemented by additional data from global databases and country reports on macroeconomic, health financing, demographic, and RMNCH outcome data as needed. It also examines the effect of other contextual factors presented in the case studies to help interpret health-financing data.

**Results:**

Dramatic increases in health funding occurred since 2000, where the MDG agenda encouraged countries and donors to invest more resources on health. Most low-income countries relied on external support to increase health spending, with an average 20–64 % of total health spending from 2000 onwards. Middle-income countries relied more on government and household spending. RMNCH funding also increased since 2000, with an average increase of 119 % (2005–2010) for RMNH expenditures (2005–2010) and 165 % for CH expenditures (2005–2011). Progress was made, especially achieving MDG 4, even with low per capita spending; ranging from US$16 to US$44 per child under 5 years among low-income countries.

Improvements in distal factors were noted during the time frame of the analysis, including rapid economic growth in Ethiopia, Peru, and Tanzania and improvements in female literacy as documented in Malawi, which are also likely to have contributed to MDG progress and achievements.

**Conclusions:**

Increases in health and RMNCH funding accompanied improvements in outcomes, though low-income countries are still very reliant on external financing, and out-of-pocket comprising a growing share of funds in middle-income settings. Enhancements in tracking RMNCH expenditures across countries are still needed to better understand whether domestic and global health financing initiatives lead to improved outcomes as RMNCH continues to be a priority under the Sustainable Development Goals.

**Electronic supplementary material:**

The online version of this article (doi:10.1186/s12889-016-3403-4) contains supplementary material, which is available to authorized users.

## Background

Leading up to the deadline of the Millennium Development Goals (MDGs), Countdown to 2015 (Countdown), http://www.countdown2015mnch.org/, engaged with several country-based teams to produce case studies by using evidence to evaluate countries’ experiences in improving reproductive, maternal, newborn, and child health (RMNCH) outcomes, highlighting achievements made towards key health MDGs, shortcomings, and recommendations on ways forward. The case studies provide an in-depth understanding of the causes and processes that led to, or detracted from the achievements of MDG 4 (reduce under-5 mortality rate by two-thirds from 1990 to 2015) and MDG 5 (reduce maternal mortality ratio by three-quarters from 1990 to 2015 [1]. Analysing how health financing is related to RMNCH outcomes was a key component of these case studies.

Countdown case studies were conducted in three phases. This paper focuses on the second phase of case studies (Afghanistan, Ethiopia, Malawi, Pakistan, Peru, and Tanzania) because they have comprehensive health financing analyses; not conducted in the first phase (Niger and Bangladesh). The third phase of case studies (China and Kenya) were not complete at the time of this study. The first paper of the Countdown case study supplement [1] provides details on the country selection criteria for the three phases of case studies. Among the 6 Countdown case studies in this analysis, Malawi, Ethiopia, Afghanistan, and Tanzania are low-income countries (LICs), Pakistan is a lower-middle income country (LMIC), and Peru is an upper-middle income country (UMIC) [2]. Focus of the case studies vary across country, where some focused on only MDG 4 while others focused on MDG 4, MDG 5, and beyond (Table 1).

**Table 1:** Country case study focus by Millennium Development Goal (MDG)

	Country	Case Study Focus
1	Malawi	Achievement of MDG 4
2	Ethiopia	Achievement of MDG 4
3	Afghanistan	Achievements MDG 5, and progress towards MDG 4
4	Peru	Achievement of MDG 4 and nutrition indicator for MDG 1, and progress towards MDG 5. More focus on MDG 4 and MDG 1
5	Tanzania	On track for MDG 4 and insufficient progress for MDG 5
6	Pakistan	Progress to achieve MDG 4 and MDG 5

The first comprehensive review of the MDGs was produced in 2005 by the United Nations, and consisted of assessing progress for the world as a whole and for various regional country groupings. Findings from this evaluation demonstrated that progress towards MDG 4 and MDG 5 was slow – especially among Sub-Saharan Africa and Southern Asian countries [3]. An upsurge in global attention following the 2005 review led to considerable increase in financial contributions to fight poverty and for countries to provide “immediate support” towards impact-focused initiatives for health [4].

Previous empirical work assessing linkages between public health spending and improvements in RMNCH outcomes are mixed. Several studies have demonstrated that an increase in public health spending significantly reduced infant and child mortality [5–8]. For example, a one-percentage point increase in the share of public health spending in gross domestic product (GDP) led to a 0.18 % reduction in child mortality [5]. On the other hand, other studies show the impact of public health spending on under-5 and infant mortality rates to be quite small and statistically insignificant, with socioeconomic and cultural factors being more influential on RMNCH outcomes [9–11]. This paper draws on data and findings from the case studies to understand how health financing contributes to progress made towards MDG 4 and 5 among selected countries, as part of the Countdown case study supplement. This will be done by answering a series of questions around trends in general health and specific RMNCH spending, influences of the different sources of funding, and the relationship between health spending and MDG progress.

## Methods

The health financing component of the Countdown case studies uses data obtained from health resource tracking tools (such as public expenditure tracking surveys, public expenditure reviews, etc. that can include frameworks, methods, and data systems for collecting and analysing data on the flow of health funds [12]), as well as other national surveys, desk reviews of key financing documents, and in some case studies, semi-structured interviews or focus group discussions were conducted in order to enhance the quantitative findings. A uniform methodology for the health-financing component of the case studies was not applied in order to cater to specific health financing questions relevant for each case study. This depended on country context, specific MDG focus, and available data. A health-financing guide was developed to assist country teams to develop the health-financing component. This is provided in the Additional file 1. The methods used for each case study are highlighted in Table 2.

**Table 2:** Health finance analysis methods for each country case study

Country	Health Finance Methods	
	Data Sources	Analyses
Malawi	Primary Data • 41 semi-structured interviews Secondary Data • National Health Accounts • IFMIS (Gov’t health expenditure) • Geocoded Malawian Aid Management Platform available from AidData (external health expenditure data) • Integrated HH Survey (for population)	Analysis Time Period 2006–2011 Qualitative and quantitative analysis at national and district levels by gov’t and development partners for 2010/11
Ethiopia	Secondary Data • National Health Accounts • Health care financing and related documents	Analysis Time Period 1995–2011 Qualitative and quantitative analysis at national to examine trends and levels in total and child health expenditures
Afghanistan	Secondary Data • Afghanistan Health Surveys • National Risk and Vulnerability Assessment Surveys	Analysis Time Period 2005–2012 Trends in MCH use and spending, and analysis at national and district levels
Peru	Secondary Data • Data from Ministry of Economy and Finance • National Health Accounts • Official development assistance data from Organisation for Economic Co-Operation and Development’s Creditor Reporting System (OECD-CRS) • Countdown database	Analysis Time Period 2000–2013 Qualitative and quantitative analysis at national and departmental levels for trends of RMNCH expenditures, and individual and group discussions to identify possible underlying factors influencing RMNCH expenditure variation
Tanzania	Secondary Data • National Health Accounts • Official development assistance data from Organisation for Economic Co-Operation and Development’s Creditor Reporting System (OECD-CRS)	Analysis Time Period 2002–2010
Pakistan	Secondary Data • Household Integrated Economics Surveys (HIES) (1998–2010) • Pakistan Social and Living Standard Measurement Surveys (PSLM) (2004–2010) • Public Sector Development Plans (PSDP) (2003–2010) • Appropriation Accounts (AA) (2006–2010) • National Health Accounts (NHA) (2008–2012) • Official development assistance data from OECD-CRS • Published scientific and grey literature	Analysis Time Period 2001–2010 Qualitative and quantitative analysis at national for trends of RMNCH expenditures, and expert panel to provide local cost estimated for MNCH services, review national guidelines, and classify vertical primary care programs

This paper descriptively analyses data from six Countdown case studies [13–18]. Questions that the analysis explores, in order to understand how health financing contributes to progress made towards MDG 4 and 5, are:

1. Did total health spending substantially increase over the MDG time period?
2. Did RMNCH spending increase significantly over the MDG time period?
3. What are the key sources of health funding and how do they vary by country?
4. What are the differences in allocation shares to RMNCH across countries and how are they related to the different rates of progress toward RMNCH outcomes?

We assessed trends in total health and RMNCH financing, health spending adjusted for population levels, spending levels by source of funding compared with total health spending, and the relationship between per capita spending levels and RMNCH outcomes to answer these questions. Total health and RMNCH financing includes government, external, household out-of-pocket (OOP), and other private financial resources. Microsoft Excel was used to generate scatter plots and graphs, and simple calculations were used to determine percent change of health financing trends. (Additional file 2 presents a table for each country with key total health and RMNCH financing data used in this paper.) This analysis also examines other factors, such as political and economic context, presented in the case studies that may also determine observed health financing trends. More details about these non-financing determinants are further explored in the first paper in this supplement [1].

Additional data was extracted from global databases and country reports including the World Bank’s World Development Indicator for macroeconomic and health financing data (for Countdown countries, not including the six in this study) [2, 19], UNICEF’s and WHO’s country statistics on maternal and child mortality rates [20–22], countries’ National Health Accounts [23–37], and National Statistics Office [38]. This was to fill any missing health outcome and financing data not reported in the Countdown case studies for Malawi, Pakistan, Afghanistan, and Tanzania. All finance data was converted to constant US$, for ease of comparability to heath financing indicators and other countries, using consumer price index factor with a base year of 2012. Health finance data sources where expressed in financial years (e.g., 2010/11), which is usually the case with NHA data, and were reported as the latter year for simplicity. For example, Tanzania’s most recent NHA expenditure data is from 2009/10, and is reported as year 2010.

RMNCH expenditures measurement is based on the reproductive and child health subaccounts definitions and consistent across five of the six Countdown case studies. This is the case for Tanzania, Malawi, Ethiopia, Peru, and Afghanistan (only for reproductive health, child health subaccount was not done). Reproductive health includes family planning and maternal health services, including postnatal care for the mother and newborn (or care up to 6 weeks after birth). Child health includes services or activities provided to children aged 0–5 years. Concluding that these two health-financing categories are not mutually exclusive. This issue will likely lead to overestimate RMNCH expenditures as a percent of total health expenditures for each country, but could not be addressed. For Peru, RMNCH per capita spending was estimated slightly different by the case study team with reproductive spending separate from maternal and neonatal estimates. This affects the comparison of per capita estimates across countries with maternal and neonatal health (MNH) per pregnant woman provided for Peru instead of reproductive, maternal, and neonatal health (RMNH) spending per woman of reproductive age – as in the case for Afghanistan, Tanzania, Malawi, and Ethiopia. The RMNH spending per woman of reproductive age (15–49) for Malawi was estimated using the NHA total RMNH spending and population estimates sourced from the National Statistics Office [38] of woman aged 15–49 from 2003–2012. The Pakistan case study team estimated maternal, newborn, and (MNCH) spending using several data sources where MNCH were not disaggregated [18]. This was done because RMNCH subaccounts were not conducted for Pakistan’s NHAs.

## Results

### Did total health spending substantially increase over the MDG time period?

**Fig. 1:**
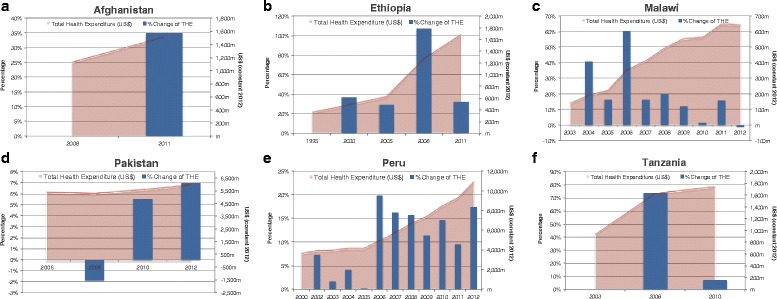
Trend and percent change in total health expenditure (THE) by country. **a** Afghanistan. **b** Ethiopia. **c** Malawi. **d** Pakistan. **e** Peru. **f** Tanzania

All six countries experienced an increase in total health expenditure (THE), although the percent change over time was more variable (Fig. 1). Malawi experienced a rapid increase in THE of US$134 million to US$638 million from 2003 to 2012, a 346 % increase. Both Ethiopia and Peru saw an increase in THE of more than 200 % after 2000. Tanzania had a lower increase in THE from US$930 million in 2003 to US$1,736 million in 2010, an overall 87 % increase. Both Afghanistan and Pakistan saw a smaller increase of 35 % (2008–2011) and 11 % (2006–2012), respectively.

Most countries also experienced an increase in per capita health spending (see Additional file 2). Malawi’s per capita health spending increased by 110 % from 2003–2012, but fluctuated from US$19 in 2003, peaking at US$41 in 2009, and then decreasing slightly to US$39 by 2012. Ethiopia’s per capita health spending increased by 244 % from 1995–2011, although it was still quite low at US$20 by 2011. Tanzania’s per capita health spending increased by 65 % from US$26 in 2003 to US$43 in 2010. Afghanistan increased slightly between 2008–2012 by 24 %, and is higher compared to the other lower-income case study countries with a per capita spending of US$56 in 2012. Peru’s per capita health spending increased at a similar rate as Ethiopia (249 % from 2000–2012), however being an UMIC the per capita health spending is substantially higher at US$331 by 2012. Pakistan was the only country within this study that experienced a slight decrease in per capita health spending between 2008 and 2012 (US$37 to US$35, a 7 % decrease).

The majority of the 6 countries’ health spending in 2010 was below the average of other Countdown countries health spending not showcased in this study, by income level (LIC, LMIC, and UMIC) (Additional file 3). Tanzania and Afghanistan are slightly above the average for LIC Countdown countries of US$38. However, Afghanistan, Malawi, and Tanzania are above the average health spending relative to GDP for LIC Countdown countries (6.7 %), while Ethiopia was slightly below at 5 % of GDP for total health spending in 2010. Pakistan and Peru per capita spending was slightly below the average for LMIC (US$87) and UMIC (US$391) Countdown countries, respectively. Although Peru’s total health spending as a proportion of GDP was consistent with the average spending for UMIC Countdown countries at 5 %.

### Did RMNCH spending increase significantly over the MDG time period?

**Fig. 2:**
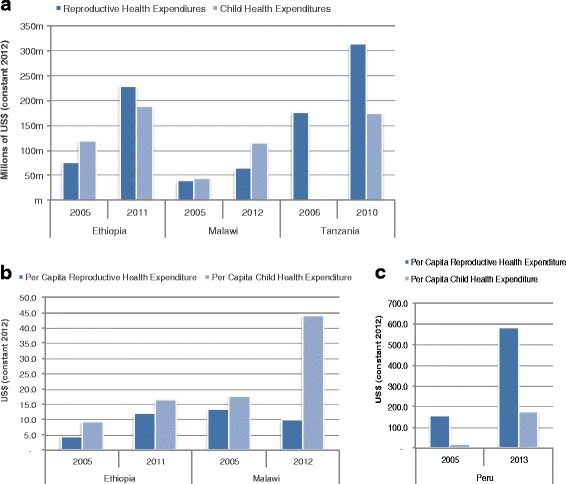
Trend of reproductive, maternal, newborn and child health (RMNCH) expenditures for Ethiopia, Malawi, and Tanzania (constant 2012 US$). **a** Reproductive, maternal and child health expenditures by country (Ethiopia, Malawi, and Tanzania). **b** Reproductive and maternal health spending per woman of reproductive age and child health expenditure per child under-5 years for Ethiopia and Malawi. **c** Reproductive and maternal health spending per woman of reproductive age and child health expenditure per child under-5 years for Peru. Note: Peru’s per capita reproductive, maternal, newborn and child health (RMNCH) expenditures are substantially higher and with all three (Ethiopia, Malawi, and Peru) graphed together, the changes in per capita reproductive, maternal, newborn and child health (RMNCH) expenditure at 2005 and after would not be as visually noticeable

RMNCH spending increased substantially across the case study countries after the agreement of the MDGs and since its first comprehensive review in 2005. Total child health (CH) expenditures substantially increased after 2005 by 58, 173 and 490 % for Ethiopia, Malawi, and Peru, respectively (Fig. 2). The trend in total CH expenditures for Tanzania and Afghanistan was not available, although there is evidence that external spending on CH substantially increased [13, 14]. Total MNH (Peru) and RMNH (Malawi, Ethiopia, and Tanzania) health spending also substantially increased after 2005 by 65, 202, 77 and 200 % for Malawi, Ethiopia, Tanzania, and Peru, respectively. In Pakistan, total MNCH expenditures increased by 96 % between 2001 and 2010 [18].

All of the countries experienced an increase in health spending per child under 5-years and woman of reproductive age (15–49) or pregnant woman (Fig. 2). For Peru, CH expenditures per child under 5 years increased from US$6 to US$176 from 2000 to 2013; a more than 20 fold increase [17]. For other countries, the increase in CH per capita expenditures was not as dramatic but still substantial. In Malawi, health spending per child was 3 times higher in 2012 compared to spending levels in 2003 while Ethiopia almost doubled its spending per child between 2005 and 2011. Peru’s MNH spending per pregnant woman increased on average almost 50 % annually (or about an annual average increase of US$42) going from US$31 in 2000 to US$574 in 2013. Consistent growth in RMNCH funding in Peru was attributed to empowering sub-national governments to participate in design, implementation, and monitoring of RMNCH interventions through decentralisation; an uptick of successful anti-poverty programs with explicit focus on RMNCH such as JUNTOS (conditional cash programme with utilisation of maternal and CH services as a condition); and the empowerment of civil societies that were instrumental to set the RMNCH agenda and ensure substantial funding to RMNCH activities despite changes in political leadership [17]. Ethiopia’s RMNH spending per woman of reproductive age increased from almost US$5 in 2005 to just under US$13 in 2011 – an increase of 182 %, albeit still low in absolute terms. Malawi experienced an overall 24 % increase from 2003 (US$14) to 2012 (US$18); a slight decline occurred between 2011–2012 of 18 % with per woman of reproductive age spending at US$22 in 2011. In Pakistan per capita MNCH expenditure increased by 67 % between 2001 and 2010 (US$4 to US$7, respectively). RMNH spending per woman of reproductive age was not available for Tanzania.

**Fig. 3:**
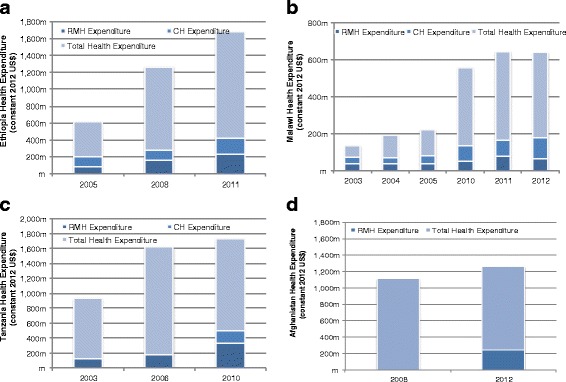
Total reproductive, maternal, newborn and child health (RMNCH) expenditure relative to total health expenditure for Ethiopia, Malawi, Tanzania, and Afghanistan (constant 2012 US$). **a** Ethiopia. **b** Malawi. **c** Tanzania. **d** Afghanistan

RMNCH spending as a proportion of THE did not always increase over time for Ethiopia, Malawi, and Peru (Fig. 3). For Ethiopia, CH spending as a percent of the THE declined from 2005 (19 %) to 2011 (11 %), while RMNH spending as a percent of THE increased slightly during the same time period from 12 to 14 %. RMNH spending as a proportion of THE also increased for Tanzania between 2003–2010 from 14 to 18 %. In Malawi, RMNH spending as a proportion of THE decreased from 19 % in 2003 to only 8 % in 2010, and then increased to 14 % by 2012. Child health expenditures as a percent of THE in Malawi declined from 17 % in 2003 to 14 % in 2011 and then rapidly increased to 18 % by 2012. In Afghanistan, RMNH spending was 16 % of THE in 2011.

Only external RMNCH spending data was available for Pakistan. External expenditures on CH decreased substantially from US$7.5 million in 2006 to US$4.6 million in 2012. Maternal health expenditures from external resources was more variable during the MDG timeframe, with US$2.5 million in 2006 jumping drastically to US$49.9 million in 2010 and then declining to US$7.2 million by 2012. This data was pulled from the Pakistan NHA, while official development assistance (ODA) data from the Creditor Reporting System (CRS) by the Organisation for Economic Co-operation and Development (OECD) shows external spending on MNCH to be substantially higher [18].

### What are the key sources of health funding and how do they vary by country?

**Fig. 4:**
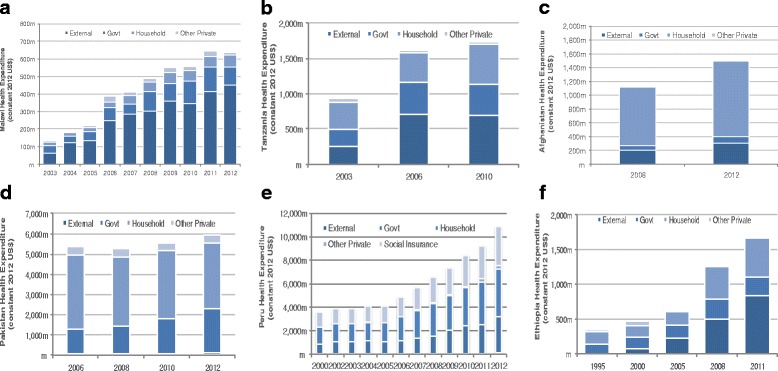
Total health expenditure by funding sources by country (constant 2012 US$). **a** Malawi health expenditure. **b** Tanzania health expenditure. **c** Afghanistan health expenditure. **d** Pakistan health expenditure. **e** Peru health expenditure. **f** Ethiopia health expenditure

Key sources of health funding vary across the six countries, and according to their income levels. Ethiopia and Malawi heavily rely on external contributions to the health sector compared to the other 2 LICs in this study, accounting for 50 % or more of total health expenditures, with increases in total health spending levels associated with increases in external aid (Fig. 4). This high level of external support may not continue as these countries develop along with competing priorities for resources especially with the new Sustainable Development Goals (SGDs) consisting of 17 goals and numerous targets compared to the 8 MDGs. Afghanistan’s health spending relied more on OOP spending, with an average of 74 % of total health spending from 2008–2012 compared to external support at 20 % of THE. For Tanzania, total spending levels increased from both government and external contributions, although a small increase occurred for health expenditures in 2010 when OOP contributions increased.

External aid is less influential on health spending levels among the middle-income countries studied. Household OOP spending on health is the main financing source for the health sector in Pakistan, consisting of 55 % of total health spending by 2012. Government spending levels is also quite high for Pakistan compared to other countries at 37 % in 2012; with an increase in the level of government health spending that corresponds with higher total health spending levels mainly between 2008 and 2012. In the case of Peru, OOP spending for health was 39 %, government health spending was 26 %, and social insurance (consisting of both OOP and government health expenditures) was 33 % of total health spending on average between 2000–2012. External and other private contributions were only 2 % of total health spending during the same time. Little funding from external resources among these middle-income countries is consistent with one criterion of external support toward countries that are typically LICs and thus more resource constraint.

**Fig. 5:**
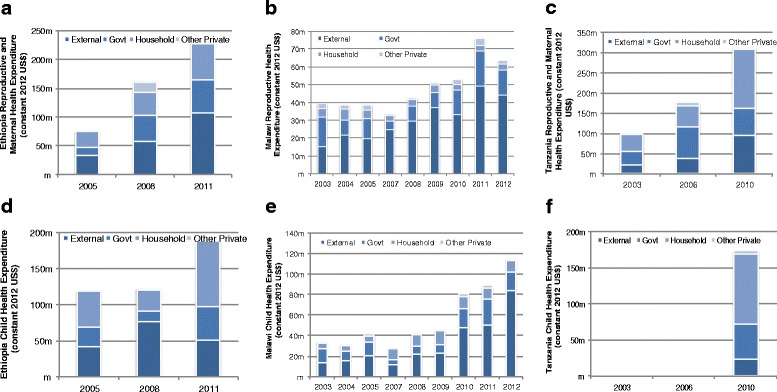
reproductive, maternal, newborn and child health (RMNCH) expenditure trend by funding sources for Ethiopia, Malawi, and Tanzania (constant 2012 US$). **a** Ethiopia reproductive and maternal health expenditure. **b** Malawi reproductive and maternal health expenditure. **c** Tanzania reproductive and maternal health expenditure. **d** Ethiopia child health expenditure. **e** Malawi child health expenditure. **f** Tanzania child health expenditure

Referring to Fig. 5, the source of funding for specific RMNCH expenditures is more mixed and specific patterns of funding sources by country income level are not as evident. Malawi continues to be heavily dependent on external resources (e.g., 73 % of total CH spending came from external donors by 2012), and a rise (or fall) in RMNCH spending depends on the increase (or decrease) in external funding for RMNCH. This seems to be the case in Ethiopia for RMNH, although CH spending levels fluctuate more according to OOP health spending levels. Donor support for CH spending in Ethiopia was 27 % and government contributed 25 % of total CH spending, while OOP was 48 % in 2011. Tanzania relies more on OOP spending for RMNCH activities, with 56 % of CH spending coming from OOP and almost 30 % from government sources and only 13 % from external sources. It is important to note that while maternal and child health services are to be free for everyone in both Ethiopia and Tanzania, OOP remains a major funding source for CH expenditures in these two countries. Donor support for CH spending in Ethiopia was 27 % and government contributed 25 % of total CH spending in 2011, while in Tanzania donor support was slightly lower at 13 % and government contributing almost 30 % of total CH spending in 2010.

### What are the differences in allocation shares to RMNCH across countries and how are they related to the different rates of progress toward RMNCH outcomes?

Increase in health and RMNCH financing was accompanied by a reduction in maternal mortality rates (MMR) and under-5 child mortality rates (U5MR), and for some of the case study countries, an achievement of MDG 4. In most cases, an increase in RMNCH spending was accompanied by a reduction in MMR (Malawi and Tanzania) [13, 15] and U5MR (Ethiopia and Malawi) [15, 16] during the same timeframe. However other contextual factors, in addition to increased health financing, may have also contributed to a reduction in MMR and U5MR to the necessary levels for MDG progress and achievement.

**Fig. 6:**
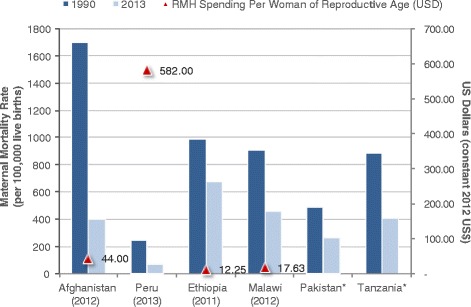
Maternal mortality rates and percent decline from 1990–2013 with most recent maternal health per capita spending by country. * No per capita reproductive health expenditure data is available. Note: Parentheses under countries is the most recent year with maternal health expenditure data

None of the six case study countries achieved the MDG 5 target, although Afghanistan, Peru, and Ethiopia are very close to achieving this goal as of 2015 with more than 80 % progress towards MDG5a [1]. Afghanistan has the highest spending per woman of reproductive age (US$45), among the LIC case study countries (where data was available), while Ethiopia is very low at only US$12 (Fig. 6). Peru has the highest spending per pregnant woman across all countries, US$574, although this estimate does not include reproductive health spending and therefore also has a different denominator compared to the other countries in this study. Most notable changes of coverage rates for key maternal health interventions in Afghanistan were skilled attendants for antenatal care (16 % in 2003 to 53 % in 2012), skilled birth attendants (14 % in 2003 to 46 % in 2012), and facility births (13 % in 2003 to 39 % in 2012) [14] – likely key drivers of maternal health spending and maternal health improvements. Likewise, Peru has increased substantially the coverage of maternal health interventions, including the percentage of women with at least 4 antenatal care visits (from 69 % in 2000 to 95 % in 2013) and skilled birth attendance (from 59 % in 2000 to 89 % in 2013), with greater progress achieved in rural areas and in the poorest quintile [17].

Sound and stable policy commitments on health investments and economic growth were listed as possible explanations for achieving substantial progress towards MDG 5 in Afghanistan, Ethiopia, and Peru [1, 14, 16, 17]. All three countries’ focused efforts on the basic health package consisting largely of maternal and child health services and substantial deployment of the health work force such as community midwives in Afghanistan and health extension workers in Ethiopia [14, 16]. In the case for Afghanistan, another explanation is that MMR was very high (1,700 per 100,000 live births in 1990) after years of conflict and no health care system, large improvements were possible with an increased focus on RMNH and health system structure. On the other hand, in settings where mortality is not as high, it is more challenging (and more expensive) to reduce MMR.

Implementation and health system constraints were noted for countries in this study for not achieving MDG 5 [1]. In the case of Tanzania, irregular implementation of key maternal health interventions or services (e.g., antenatal care and clean birth practices) over the decades slowed progress towards MDG 5 [13]. In Ethiopia several possible reasons for not achieving MDG 5 include lack of strong referral linkages between health facilities and deep-rooted cultural practices or beliefs that lead to underutilisation of services such as institutional delivery [16]. In Pakistan an equity analysis revealed pro-rich utilisation of government health facilities for antenatal care, institution based obstetric deliveries and postnatal care during 2001–2010 [39].

**Fig. 7:**
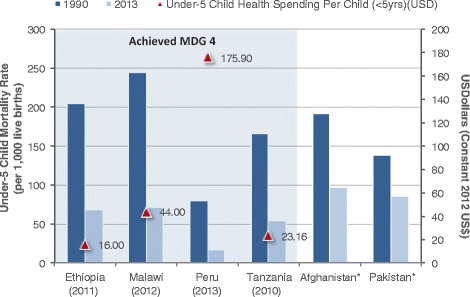
Percent decline for U5MR and health spending per under-5 child by country. *Per capita child health expenditure data is not available. Note: Parentheses under countries is the most recent year with child health expenditure data. Countries within the blue area achieved MDG 4.

Four out of the six countries in this study met MDG 4 – Peru, Malawi, Ethiopia, and Tanzania. The amount of spending per child under 5 years varied greatly (Fig. 7). Ethiopia spent the least amount with only US$16 per child in 2011, while Peru spent the most at US$176 in 2013. It was found that a decline in child mortality in Ethiopia corresponds with the rapid increase in total health spending due to the implementation of health and health financing programs, policies, and strategies including the Health Extension Program (2004), Health Care and Financing Strategy (2005), and Child Survival Strategy (2005) [16]. High political commitment to the health sector also led to streamlining a majority of domestic and external resources for health through a harmonisation initiative, which focuses health resources jointly to attain common targets and goals [1, 16]. Malawi spent US$44 per child in 2012, while Tanzania spent US$23 per child in 2010. In Pakistan, government health facilities were found to be pro-poor for CH services during 2001–2010, yet targets on immunisation coverage remained a big challenge throughout the decade of 2000 [39]. Pakistan did not achieve MDG 4 despite public facilities being pro-poor and an increase in MNCH financial resources.

Other factors, in addition to health spending on CH services, may explain reductions in U5MR and IMR. Increases in GDP per capita have been associated with significantly reducing U5MR and IMR [40]. A number of the Countdown case study countries experienced rapid economic growth during the MDG era (see Additional file 4). Since 2003 Ethiopia experienced more than 10 % real annual GDP growth (equating to an annual GDP per capita annual increase of 8 %), one of the fastest growing economies among Sub-Saharan African countries [16]. Tanzania experienced a 7 % annual increase in real GDP (or 4 % annual increase in real GDP per capita) since 2000. The Tanzania case study also found that an increase in gross national income (GNI) per capita was significant, although a weak association, in reducing U5MR [13]. Additionally, improvements in female literacy has led to a reduction in child mortality [40, 41], and Malawi case study identified that this may have contributed to increased child survival [15].

## Discussion

Dramatic increases in health funding occurred among all countries in this study since 2000, with increased focus on the MDGs. Low-income countries relied substantially on external support in order to rapidly increase health spending, something that will need to continue in the near future to achieve the SDGs, but brings concerns of fiscal sustainability. Conclusions on whether this increase in external support has led to a displacement of government health spending as found in previous literature [42–44] could not be concluded on such few observations. The dependency on external resources is much less among the studied middle-income countries, with a majority of the health funding coming from government as well as OOP health spending. Some studies highlight that high OOP health spending is regressive by exposing them to potential catastrophic spending [45–47]. An 89 cross-country study found that catastrophic health spending and impoverishment remained high where OOP spending on health that was more than 15–20 % of total health spending [47]. Pakistan, Afghanistan, and Peru all have substantially higher OOP spending than this threshold in 2012 of 55, 73 and 37 %, respectively. Exploration into the causes and economic impact of the high OOP spending among the case study countries, especially for Afghanistan, a LIC, is needed.

**Fig. 8:**
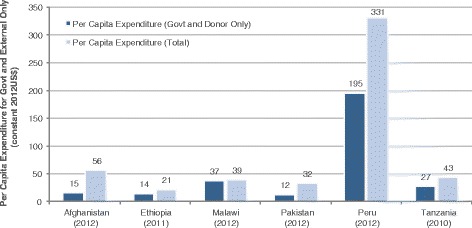
Per capita health spending by country for most recent year (constant 2012 US$)

The High Level Taskforce on Innovative International Financing for Health Systems (HLTF) was set up in 2008 to identify innovative and additional financing to strengthen health systems among 49 of the lowest-income countries. In 2009, the HLTF modelled the per capita resource requirements for providing a basic health service package among LICs. By using the WHO normative costing approach, it was estimated that countries should spend US$54 per capita (2005 constant) in order to deliver all necessary health services. This translates to US$86 per capita in 2012 terms for government and donor funding to ensure basic primary health care services within LICs [48]. None of the low-income countries in this study have reached this estimated level of per capita spending (Fig. 8). Afghanistan, Ethiopia, Malawi, and Tanzania, had a total health per capita spending from government and donors of US$15 (2012), US$14 (2011), US$37 (2012), and US$27 (2010), respectively; implying a financial gap between US$49-US$74 of per capita spending. It is unlikely that these four countries will close this gap in the near future given historical trends of health spending.

RMNCH funding also substantially increased across all studied countries, especially after the first review of the MDG progress in 2005. Contributing factors that led to an increase in RMNCH funding and improvements in RMNCH outcomes are political stability; consistent political commitment to health; rapid economic growth; engagement with community; decentralisation; anti-poverty programmes with explicit focus on RMNCH; and, for some LICs, increased external support [1, 13–18]. Malawi RMNCH financing is heavily reliant on external support, consisting of 70 % or more of total RMNCH funding in 2012, along with Ethiopia’s RMNH funding comprising of 47 % from external support in 2011. High OOP spending for RMNCH activities in Tanzania and CH activities in Ethiopia are of particular concern given that maternal and child health services are to be free for everyone. Possible explanations might be heavy reliance on private pharmacies for drugs and supplies when public health facilities are out of stock, imposing costs on the individual or individuals using private providers for CH services that may be closer to home or perceived better quality of services [49–51].

Substantial progress was made toward MDG 4 and 5 among some countries, even with per capita RMNCH spending below $50. One possible explanation for this finding is that health spending was targeted toward effective RMNCH interventions. The Lives Saved Tool (LiST) analysis conducted under the Countdown case studies demonstrates that certain interventions were particularly effective in reducing child mortality rates. For example, almost half a million of children’s’ lives were saved in Ethiopia due to key interventions implemented from 2000–2011; 44 % of which was due to activities that focused on reducing stunting [16]. On other hand, resource inefficiencies may be contributing factors that detracted Pakistan’s ability to achieve MDG 4 and 5. Such inefficiencies were found with duplication of program implementation and routine PHC services, along with an overlap of human resource roles between health workers such as the lady health workers, lady health visitors, and community midwives [18]. The possibility that countries with low RMNCH per capita spending were able to achieve MDG 4 or make substantial progress towards MDG 5 by targeting health spending towards more effective RMNCH interventions needs to be tested. Understanding the determinants behind such successful cases could provide a way forward for achievements during the post-MDG era.

While the Countdown case studies were not designed to test causal relations between financing and RMNCH outcomes, the studies show that reductions in MMR, IMR and U5MR were accompanied by an increase in RMNCH financing. Improvements in other distal factors were noted to have potentially contributed to reducing maternal and child mortality rates in the case studies such as rapid economic growth in Ethiopia, Peru, and Tanzania and improvements in female literacy as documented in Malawi for improved child survival. Other studies have shown that public spending leads to a reduction in infant and child mortality, albeit a small one [5–9].

Two main limitations of this study are the availability of regularly reported RMNCH expenditure data across the countries and findings presented are not generalisable. Inconsistency in regularly reported RMNCH expenditure data across countries, along with the defined scope of the case study (Table 2), did not allow for a more uniform methodology to conduct the health finance component of the case studies. Not every case study focused on the achievements, progress (or lack thereof) on both MDGs 4 and 5. Moreover, the time periods of focus for the health financing section of the case studies was dependent on health expenditure data availability. For example, Malawi’s period of focus was 2006–2011; Ethiopia’s was 1995–2011, while Pakistan’s was 2001–2010. Data sources used were also mixed, although when available most used NHA data. At times, these data were also supplemented by other country-specific data sources, such as the Household Integrated Economics Surveys for OOP spending estimates for Pakistan and data from the Ministerio de Economía y Finanzas for MNH and CH data for Peru, while others (Pakistan, Peru, and Tanzania) used the OECD-CRS database for external contributions to RMNCH. As a result, the case studies are purely descriptive. Robust econometric analyses to understand the causal relationship between levels and sources of health financing and RMNCH outcomes were not feasible because of inconsistent and limited health and RMNCH expenditure data with a small sample size of only 6 countries. Thus, these findings are not generalisable toward other LICs, LMICs, and UMICs experiences with health financing and RMNCH progress during the MDG era.

While RMNCH expenditure tracking efforts have improved since 2000, such as the inclusion of sub-accounts under the NHA, comprehensive and consistent RMCNH expenditures for many countries is still lacking. Resource tracking tools are used to collect and analyse health expenditure data within countries but many are not institutionalised (conducted on a regular basis), accounting methods for RMNCH expenditures may not be mutually exclusive, or face limitations because of inconsistent or subjective methods used over the years. For example, the government spending for the reproductive and child health subaccounts for Ethiopia are based on assumptions developed from background materials (such as health service reports) and *expert opinions* [34, 35]. This leaves government estimates of RMNCH expenditure prone to estimation errors due to the use of different accounting assumptions from 1 year to the next. Therefore, identifying time trends in RMNCH expenditures comes with caveats because methods used over time for the NHA subaccounts might be different, as was the case for Ethiopia. Implementation of SHA 2011 may minimise some of these issues by tracking expenditures according to the classification of Global Burden Disease. Furthermore, the OECD-CRS provides specific data around RMNCH expenditures but this only captures donor disbursements for health. This data set does not break out the RMNCH-specific expenditures, which is done by external agencies like Countdown and IHME [52, 53]. The OECD recently added a code specifically for RMNCH funding, but this new indicator still does not disaggregate funding within RMNCH.

Global efforts to collate RMNCH expenditures into one database or report are fragmented. The Commission on Information and Accountability (CoIA) for Women’s and Children’s Health [54] began an effort to have all 75 Countdown countries to report their total RMNCH expenditure by financing source by 2015. To date, 27 countries have produced this data but only 8 out of the 27 country data were provided for the 2015 Countdown report [52]. Another source for health financing data is the WHO Global Health Expenditure Database, which attempts to collate all NHA data into one “master” database that is accessible to everyone in an open-sourced platform. A draw back in using this database is that key NHA data from the RMNCH subaccounts is not consistently available for most countries. Child health expenditure data is compiled only for Liberia in 2007 and Malawi from 2003–2005, while RMNH expenditure data is compiled for 17 countries but this data is still very limited in terms of years available in the database versus data available from the NHA reports. The WHO does provide the NHA reports for countries but requires one to manually extract the NHA data from the tables. Institutionalising or standardising reporting systems on RMNCH interventions and resource use, along with improving the information collected and provided into an accessible database, is important to monitor programs and understand health progress at the country level, and globally.

## Conclusions

Through the lens of six country case studies, key lessons are learned on trends in RMNCH financing, contributing factors to increase resources and progress towards MDGs, and gaps in information to assess the magnitude to which health financing has on health outcomes (Table 3). In the countries studied, the MDG agenda encouraged the global community and country governments to mobilise more resources for health and specifically for RMNCH. Although per capita spending on health and RMNCH in some countries are still quite low, significant progress was made toward the RMNCH MDG targets, and achievement of MDG 4 among some countries.

**Table 3:** Key messages

***Key messages***
1. Total health spending increased across the six Countdown country case studies (Afghanistan, Ethiopia, Malawi, Pakistan, Peru, and Tanzania) since 2000, where the MDG agenda encouraged countries to invest more resources in health. Malawi, Ethiopia, and Peru had the most notable increase in health spending of more than 200 % after 2000.
2. Reproductive, maternal, neonatal, and child health (RMNCH) spending substantially increased during the MDG timeframe. Since 2005, total CH expenditures increased (in constant 2012 US$) for Ethiopia, Malawi, and Peru by 58, 173 and 490 %, respectively. Total MNH (Peru) and RMNH (Malawi, Ethiopia, and Tanzania) health spending also increased substantially during after 2005 by 65, 202, 77 and 200 % for Malawi, Ethiopia, Tanzania, and Peru, respectively. In Pakistan, total MNCH expenditures increased by 96 % between 2001 and 2010.
3. No country achieved MDG 5, however Afghanistan, Ethiopia, and Peru have made considerable progress with more than 80 % of the target achieved, and variable rates of per woman of reproductive age spending of US$44, US$12, and US$582, respectively. Ethiopia, Tanzania, and Malawi met MDG 4 with US$16 (2011), US$23 (2010), and US$44 (2012) health spending per child under 5 years, respectively.
4. Common themes of contributing factors that led to an increase in RMNCH funding, and thus improvements to RMNCH outcomes, are political stability; consistent political commitment to health; rapid economic growth; community engagement; decentralisation; anti-poverty programmes with explicit focus on RMNCH; and for some low-income countries, increased external support.
5. Enhancing RMNCH resource-tracking systems will make it easier to assess where countries invest resources and identify steps toward outcome improvements.

Other distal factors, in addition to health financing, also most likely contributed to the success of achieving some of the MDGs across countries. As in the case of Tanzania an increase in GNI per capita was significantly associated in reducing U5MR [13]. Unpacking the positive influence of other factors, such as macroeconomic changes and improvements to other sectors, may have on health outcomes is complicated and beyond the scope of this paper.

RMNCH continues to be a priority with the newly created and agreed upon SDGs. However, the behaviour and patterns of global and domestic funding for the future is unknown. Continued focus on financing initiatives that lead to improved health outcomes is needed in order to ensure the continued success that was made during the MDG era. This begins with improvements to track RMNCH expenditures across countries. We look to organisations that are attempting to improve resource-tracking deficits such as the WHO with the push to implement the revised System Health Accounts (SHA2011) and the analytical work associated with World Bank’s new Global Financing Facility, a key-financing platform of the United Nations Secretary-General’s *Every Woman Every Child* initiative [55, 56]. These efforts involve better reporting, collecting, deriving and using data (e.g. lack of spending does not necessarily mean no intervention, but maybe intervention under another classification or category). Outcomes from these current efforts remain to be seen.

This paper raises important research questions for future analysis around RMNCH financing in terms of spending structure and impact on outcomes. Have countries with low RMNCH per capita spending achieved MDG 4 and 5 by focusing health spending on more effective RMNCH interventions? Is potential domestic RMNCH funding being displaced by the increase in external support? Does health financing directly lead to improved RMNCH outcomes? What are the effects of other sectoral spending (education, agriculture, infrastructure, etc.) on RMNCH outcomes?

## Abbreviations

CH, Child health; Countdown, Countdown to 2015; CRS, Creditor Reporting System; GDP, Gross domestic product; GNI, Gross national income; HLTF, High Level Taskforce on Innovative International Financing for Health Systems; IMR, Infant mortality rate; LIC, Low income country; LMIC, lower-middle income country; MDG, Millennium Development Goal; MMR, Maternal mortality rate; MNCH, Maternal, newborn, and child health; NHA, National health accounts; OECD, Organisation for Economic Co-operation and Development; OOP, Out-of-pocket; RMNCH, Reproductive, maternal, newborn, and child health; RMNH, Reproductive, maternal, and neonatal health; SDG, Sustainable development goal; THE, Total health expenditure; U5MR, Under-5 child mortality rate; UMIC, Upper-middle income country; WHO, World Health Organisation.

## Additional files

Additional file 1: Health Financing Analysis for Countdown Case Studies: A Guide. (PDF 1459 kb) Additional file 2: Country specific health spending data tables. (DOCX 44 kb) Additional file 3: Health spending data for all Countdown countries by income status in 2010. (DOCX 219 kb) Additional file 4: GDP per capita trend data by country. (DOCX 707 kb)
