# Supplementary material for: Countdown to 2015 country case studies: what can analysis of national health financing contribute to understanding MDG 4 and 5 progress?
Source: BMC Public Health. 2016 Sep 12;16(Suppl 2):792. doi: 10.1186/s12889-016-3403-4 (PMC5025819; doi:10.1186/s12889-016-3403-4)
Supplement: Additional file 1: — Health Financing Analysis for Countdown Case Studies: A Guide. (PDF 1459 kb) [file 12889_2016_3403_MOESM1_ESM.pdf]

Health Financing Analysis for Countdown Case Studies

A Guide

12/4/2014

Prepared by Nirmala Ravishankar, Carlyn Mann, Patricia Hernández-Peña, and Ravindra Rannan-Eliya

This Guide was prepared by the Financing Working Group as a resource to assist Countdown to 2015 country case study teams design and implement health financing analyses for their case studies.

Table of Contents

1. Introduction 2
2. Health Resource Tracking 3
   2.1. Development assistance tracking 3
   2.2. National Health Accounts 6
   2.2. Other Health Resource Tracking tools 9
3. Financial Protection and Access to Services 12
4. Equity Analysis 18
5. Costing 24
6. Country Examples 27
   6.1. Peru 27
   6.2. Ethiopia 28

References 30

1. Introduction

The goal of the Countdown country case studies is to use evidence to tell a story about a country's track record in improving reproductive, maternal, newborn, and child health (RMNCH) outcomes and achieving Millennium Development Goals (MDGs) 4 and 5 to reduce child mortality and improve maternal health respectively, with a focus on explaining how progress was achieved. Analyzing health financing issues that had an impact on RMNCH outcomes can be an important component of a case study. The particular health financing questions that are relevant for a given case study depend on the country context. For example, in some countries, changes in the level and composition of health expenditure for RMNCH may be central to explaining progress made in RMNCH-related intervention coverage and health outcomes. In others, improved efficiency in the use of RMNCH funds might be the reason for improved results or changes in financing may have served to reduce inequalities in access to critical services, which in turn may have accelerated improvements in overall RMNCH outcomes. Country case study teams will need to design the health financing component of their case study accordingly.

The purpose of this guide is to assist country teams working on the Countdown to 2015 (Countdown) case studies to conceptualize and undertake the health-financing analysis for their study. It describes different types of analyses country teams could undertake, and provides links to relevant resources and tools that teams can use in developing particular analyses.

This Guide follows a menu approach, introducing and describing health financing analytical frameworks that the country team could use based on the country context. Sections 2 to 5 present the following four types of analyses that case study teams could undertake: (i) health resource tracking, (ii) analysis of financial protection and access to services, (iii) analysis of equity in public financing of health programs, and (iv) costing.[1] For each type of analysis, this Guide discusses the research questions that can be answered using that particular analytical lens, the kind of data that is needed, and relevant reference materials. In Section 6, we present summaries of the health financing analysis undertaken in Peru and Ethiopia as illustrative examples.

[1] These topics were selected based on comments received from the country teams participating at a Countdown case study meeting held in Nairobi, Kenya in February 2014.

2. Health Resource Tracking

Health resource tracking (HRT) refers to frameworks, methods, and data systems for collecting and analyzing data on the flow of health funds (Powell-Jackson and Mills, 2007). Information produced by HRT analyses on the amount of health spending, how it has changed over time, and its composition by source, purpose, disease-focus etc., is an essential ingredient for policy-making and planning at the national-level, as well as greater donor coordination at the international level. HRT information can also be used to identify bottlenecks, points of leakage, or inefficiencies in the flow of resources, and in turn improve accountability and enhance performance in the health sector.

HRT tools are varied in their scope and purpose. Some focus on expenditure from a single source, like donors, national governments or households, while others look at total spending from different sources. The analyses can also vary in terms of their geographical focus, measuring resources at the global, national or sub-national level. Some studies have measured resources for particular diseases or health areas, while others have analyzed resources for the sector as a whole.

Below, we discuss two HRT frameworks that are most relevant to the Countdown case studies since they can generate information about RMNCH-related expenditures: development assistance tracking and National Health Accounts (NHA). We also briefly describe other HRT frameworks that can provide information on health resource flows more broadly – i.e. not specific to RMNCH programs – that may be of use to case study teams to describe the overall health system context.

2.1. Development assistance tracking

Measuring and analyzing aid flows is the focus of development assistance tracking activities. In countries that receive significant amounts of aid for the health sector, exploring external assistance for RMNCH programs could be an important part of the health financing analysis component of the country case study.

Background

Data on aid flows compiled by the Development Assistance Committee (DAC) of the Organization for Economic Cooperation and Development (OECD) has served as the backbone for global development assistance tracking activities. Specifically, OECD's Creditor Reporting System (CRS), which captures project-level information on development assistance from OECD member countries, several multilateral agencies and some large private foundations, has been the single most important source of information for most international health resource tracking efforts (OECD, n.d.). The CRS tracks official development assistance (ODA), which includes both grants and loans on concessional terms. For each project, the database tracks both the amount of funding committed by the donor at the start of the activity and actual funds disbursed each year, along with a variety of descriptive information including the recipient country, type of aid (grant, loan, etc.), and purpose of the project. The aid flows are measured on a calendar year basis. OECD makes considerable effort to standardize the data reported by member countries, and the quality of the information has steadily improved.

Using data from the CRS, studies have measured the total envelope of ODA for health (Ravishankar et al*.,* 2009; Sridhar and Batniji, 2008), the relative contributions of different donors (Kindornay and Besada, 2011), ODA going to specific diseases or health areas such as RMNCH or neglected diseases (Powell-Jackson et al., 2006), as well as financing flowing to specific regions or types of countries such as aid for fragile states (Patel et al., 2009). Recent initiatives have attempted to expand the remit of ODA tracking by supplementing information from the CRS with data on aid from non-DAC donors like China and Brazil as well as contributions from private philanthropic institutions (Aiddata.org, n.d.; Ravishankar et al., 2009).

Possible Research Questions

Country case study teams can use information on ODA for RMNCH to examine the following topics and to consider if changes in ODA funding influenced improvements in RMNCH outcomes:

1. What are the overall trends in RMNCH aid flows (changes over time, RMNCH aid as a percentage of total health ODA, comparison of RMNCH aid for their country versus global trends in RMNCH aid, etc.)?
2. What is the relative role of different donors supporting RMNCH programs?
3. How much ODA do different health areas within RMNCH receive?
4. Is there year-on-year volatility in aid for RMNCH?

Potential Data Sources

While the CRS is publicly available and case study teams can access the data directly from OECD, the team would then need to identify and code the RMNCH projects. Doing this for multiple years of data would require considerable effort. A group of scholars based at the London School of Hygiene and Tropical Medicine (LSHTM) have manually re-coded all the projects in the CRS database to identify all records that are related to RMNCH. They have used this data to publish regular updates about what is happening to aid for RMNCH at the global level, as well as comparing trends in ODA for the Countdown countries versus all developing countries (Jackson-Powell et al., 2006; Pitt et al., 2010; Hsu et al., 2012). LSHTM's Countdown to 2015 database can also be used to look at RMNCH aid for a single country, as Tanzania has done in its case study. For example, Figure 2.1 shows a breakdown of the total development assistance for RMNCH in Tanzania by focus area, and how this has changed over time. Figure 2.2 shows a breakdown of maternal, newborn and child health by donor (Martinez-Alvarez, 2014). County case study teams that are interested in analyzing development assistance for RMNCH programs in their county can approach the LSHTM team to get data for their country.[2] The data is available in Excel format, and can be analyzed using pivot tables.

In addition to the CRS, countries may have developed national data systems for tracking donor flows. Such a database may either be specific to the health sector, or track development assistance across all

[2] Please email Professor Josephine Borghi at Josephine.Borghi@lshtm.ac.uk to request data for your country on ODA for RMNCH programs.

sectors. The team could also explore these data sources, especially if they are more politically acceptable to the government than international databases.

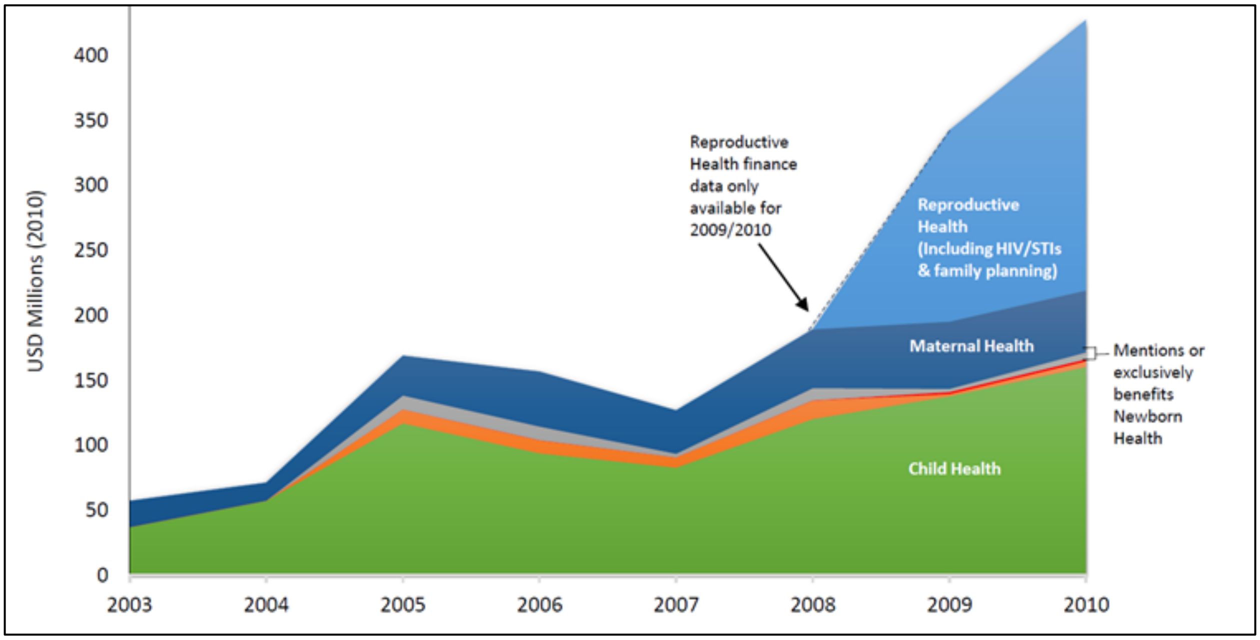

Figure 2.1: Development assistance for RMNCH in Tanzania (Source: Martinez-Alvarez, 2014)

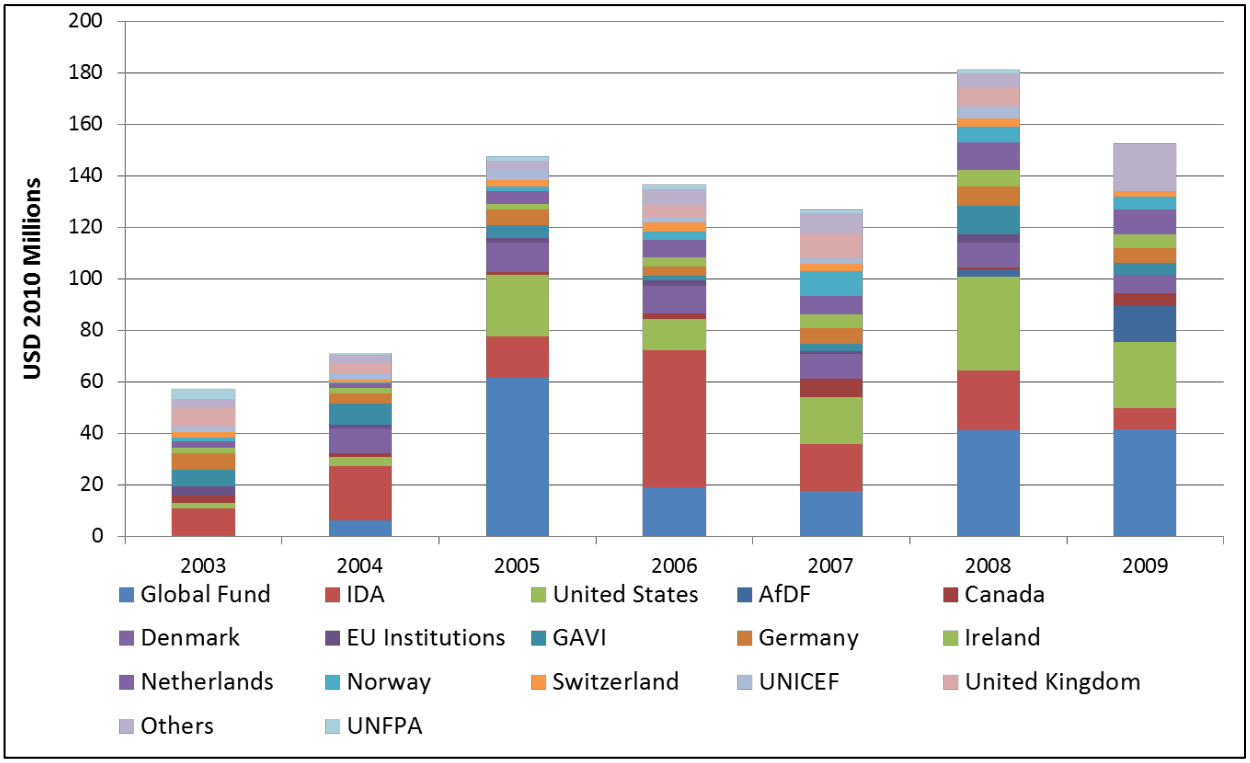

Figure 2.2: Development assistance for MNCH in Tanzania, disaggregated by donor (Source: Martinez-Alvarez, 2014)

The analysis of ODA and NHA data in the Tanzania case study

The Tanzania team assessed health resource use at the national level using two sources of data: the Countdown database and the NHA. The Countdown database was used to analyze ODA flows to RMNCH. This was done in several steps. First, the Countdown database projects relating to Tanzania (those which had Tanzania as a recipient) were extracted for each year between 2002 and 2013 to create a Tanzania-specific database. Second, a pivot table was used to calculate the totals for Maternal and Newborn Health, Child Health, Maternal and Newborn or Child Health projects mentioning newborns and projects exclusively benefiting newborn by year and by donor. In addition, for the years where Reproductive Health (RH) projects were available (2009-2012), RH was calculated by subtracting MNCH from RMNCH. The final step was to visualize the results, which was done through the use of area graphs and stacked bar charts.

NHA data were used to assess total expenditure on RMNCH-related fields by source over three points in the time period of study (2002/03, 2005/06 and 2009/2010). In particular, expenditures on HIV/AIDS (which include prevention of mother-to-child transmission (PMTCT)), reproductive health (which include maternal and newborn health and family planning) and child health were analyzed. These expenditures were extracted from their respective NHA sub-accounts. Data were then analyzed by source, including government, donor and out-of-pocket sources over the three points in time in which they were available. The results were subsequently plotted using bar charts.

2.2. National Health Accounts

NHA is a methodology that countries use to measure and categorize health spending associated with their population over a calendar or fiscal year. Since describing the composition of health spending in the country and how it has changed over time, as well as discussing what has happened to RMNCH-specific spending, if that information is available, are central to the story that the case studies are attempting to tell. NHA data can be an invaluable resource for the country case study teams. NHA data will allow the team to describe the health financing situation (both current and historical) and to identify the need for or feasibility of a financing intervention. It can also be used for more sophisticated analysis, such as can the national RMNCH situation improve by modifying current health spending mechanisms? What are the potential barriers for effective financing? Which changes in financing could lead to more effective spending? How equitable is the financing system?

Background

The NHA provides a framework for determining what expenditures should be included in the measurement of national health spending as well as detailed classifications for disaggregating the total pie of expenditure along various dimensions such as how the expenditure is financed, what services are consumed, the providers of the services consumed, disease- or intervention-focus of the expenditure, etc. In order to make NHA measurements comparable across countries and over time, OECD developed uniform guidelines and codes for measuring and categorizing different types of health spending in 2000

called the System of Health Accounts or SHA 1.0 (OECD, 2000). By 2010, nearly 130 countries around the world had used SHA 1.0 to assess domestic health spending patterns (World Bank, 2010). In 2011, OECD, Eurostat and WHO released A System of Health Accounts 2011 or SHA 2011, a revised framework for undertaking NHAs that addressed some of the limitations of the original framework and updated it to reflect new developments in health systems (OECD et al., 2011).

The concept of health consumption is central to the measurement of health expenditure in the NHA framework (OECD et al., 2011). In other words, NHA is measuring in monetary terms what health services and goods have been consumed by the population of a country over a fixed period of time, typically a year. The key quantity of interest under SHA 1.0 was total health expenditure, which included both health care consumption in the current year and as well as spending associated with the development or acquisition of health infrastructure such as health facilities and medical equipment that are consumed over multiple years (OECD, 2000). In contrast, SHA 2011 draws a clear distinction between current health expenditure and capital formation, and accounts for them separately (OECD et al., 2011).

To define what health goods and services should be counted, the NHA uses the functional approach, which focuses on the purpose of consumption activities (OECD et al., 2011). Specifically, the NHA counts as health spending the expenditure associated with all activities whose primary purpose is to promote health (including the prevention and cure of diseases, care for chronic conditions, and the administration of health programs), regardless of who funded or paid for that activity and who provided the service. For all activities included within the boundary of health consumption using the function approach, the NHA also takes into account what type of entity provided the goods or services and how the consumption was financed. This gives rise to the tri-axial system of health accounting, shown in Figure 2.3, comprised of the health consumption, service provision, and health financing interfaces (OECD et al., 2011).

Each axis is associated with accounting dimensions along which total health expenditure is disaggregated. For the financing interface, health spending is broken down by financing scheme (the mechanism through which expenditure takes place such as social health insurance, out-of-pocket expenditure, etc.), financing agent (the entity that controls the spending such as the ministry of health, insurance companies, households, etc.), and financing source (the original source of funding such as taxation, development assistance, etc.). In the health care consumption interface, health expenditure is disaggregated according to health function (the purpose for which the spending took place, such as curative care, preventive care, administration of health financing schemes, etc.), age of the beneficiaries, gender of the beneficiaries, and disease-focus. Finally, in the provider interface, health spending is broken down according to who provides the service (such as hospitals, health centers, etc.) and the factors of provision (the inputs needed to produce the service such as drugs, salaries, etc.).

While health spending as a whole is the focus of the NHA, some countries have undertaken additional sub-accounts for priority health areas like HIV/AIDS, malaria, reproductive health (RH) and child health (CH). Sub-accounts are essentially “mini-NHAs” for the health area in question, providing more detailed

break-downs of spending for that health area by financing source, agent, function, provider, *etc*. Under SHA 2011, countries are encouraged to use disease classifications to categorize health spending. In other words, they are meant to break down the total pie of health spending by disease or health priority area instead of undertaking sub-accounts for priority health areas. Countries may apply the disease classification to a portion of total spending, and gradually expand the share of spending that is classified by disease as the quality and availability of this information improves.

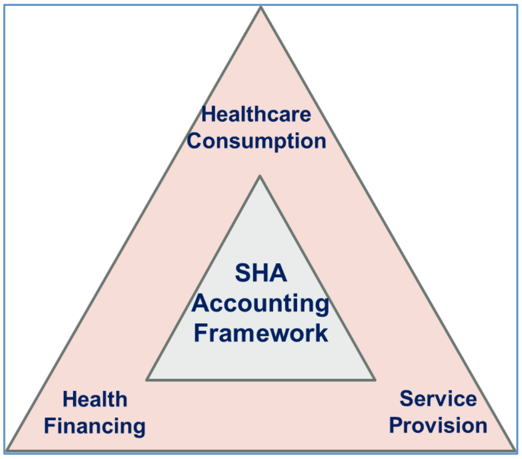

Figure 2.3: NHA's tri-axial system for conceptualizing health spending

Possible Research Questions

Country case study teams can use NHA data to explore the following topics and consider potential influences on changes in RMNCH outcomes:

1. What is the overall composition of health spending in the country in terms of key sources of health financing, functions, providers, *etc*., and how this has changed over time?
2. How much is spent on RMNCH, both in absolute terms and as a share of total spending? Is this spending increasing or decreasing?
3. How are RMNCH services financed? How are resources mobilised for RMNCH purposes and how are they managed? Are resources are pooled? How are services purchased? What is the extent of financing risk protection for RMNCH? Are individuals paying out-of-pocket for RMNCH services? Are RMNCH programs very donor-dependent?
4. In terms of different providers of RMNCH services, who accounts for the bulk of the consumption? What are the key inputs?
5. How much is spent on prevention versus cure? What population consumed the most RMNCH spending (by gender or age)?

Potential Data Sources

NHA estimation requires expenditure data from multiple sources including government budget documents, national population surveys that provide an estimate of household health spending, as well as institutional surveys of development partners, insurance companies, and employers to capture the amount they spend on health.

Undertaking an NHA is most likely not feasible within the scope of a country case study. However, teams can refer to existing NHA estimations to analyze the overall pattern of health spending in the country, which can serve as valuable background information for the case study. It is worth noting that countries are currently in the process of transitioning from SHA 1.0 to SHA 2011. This will likely impact the comparability of estimates over time, if countries do not revise earlier estimates for comparability.

In countries that have undertaken one or more rounds of RH and CH subaccounts, the NHA can be used to examine the composition of RH and CH spending in terms of the sources of financing, financing agents, the functions or interventions, and providers, as well as trends in total RH and CH spending in the country. Figure 2.4, which shows a comparison of RH spending across countries in sub-Saharan Africa, was generated using data from RH subaccounts (Nguyen et al., 2011).

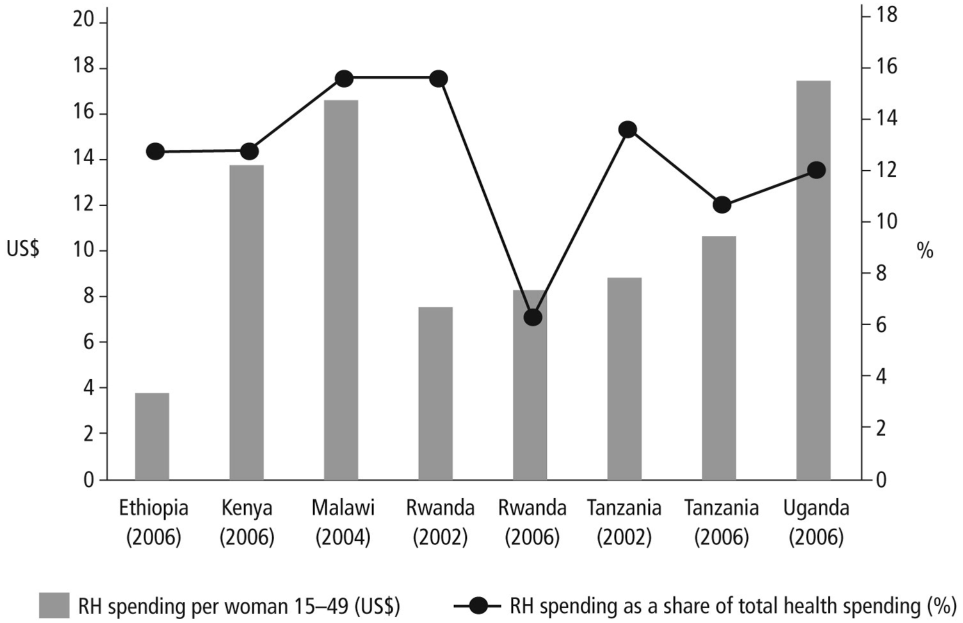

Figure 2.4: Comparison of RH spending across 6 Sub Saharan African countries (Source: Nguyen et al., 2011)

2.2. Other Health Resource Tracking tools

The HRT frameworks discussed above can provide information on resource flows related to RMNCH. Next, we mention and briefly describe other HRT tools that provide more general information about resource flows in the health sector that may be useful to the country case study teams to analyze general trends in the health sector and the policy context that serves as a backdrop for RMNCH programs. It is important to note that the Countdown case study teams are not expected to undertake such analyses. But these may serve important secondary sources of information that the team may utilize in their case study.

Public Expenditure Tracking Surveys (PETS)

PETS track the flow of funds from the national treasury through various levels of government down to the frontline providers of public services like schools and health facilities. They assess what share of

public funds allocated in the national budget for service delivery actually reaches service providers and whether the funds are spent as they are intended. The extent of unaccounted resources, estimated from a representative sample of facilities, is indicative of the magnitude of leakage or inefficiency in the system.

Information from PETS exercises has increasingly been used in conjunction with that from Quantitative Service Delivery Surveys (QSDS). While PETS focus on public spending, QSDS assess the efficiency and quality of service delivery by gathering information about the determinants of output like availability of supplies, facility management, staff absenteeism, etc. Together, the PETs and QSDS provide a complete picture of efficiency and equity in service delivery in the public sector.

Since the mid-1990s, over 50 PETS/QSDS exercises have been implemented in approximately 36 countries around the world, nearly two-thirds of which are in Africa (Gauthier and Ahmed 2012). PETS produce information that speaks to a range of issues related to governance in the health sector. In helping to identify specific problems in the flow of funds through the public sector, PETS serve a diagnostic purpose. They can go further to function as an analytical tool to ascertain the causes for the irregularities. They can additionally be used to assess the impact of key government programs or reform measures.

The most prominent output from a PETS exercise, one that easily garners the attention of stakeholders and the media, is evidence of leakage. The tracking survey commonly provides estimates of leakage in the health sector, measured by the share of resources intended for but not received by health facilities from a sample of facilities. Leakage can be caused by many factors. While corruption is the factor that draws the greatest attention, there may be other reasons for national outlays not equaling in-flows at the facility-level including incomplete records, administrative overheads, etc. PETS have also been used to investigate other issues such as delays in disbursements, the inequitable allocation of resources especially across geographical units, and the impact of decentralization. Countdown case study teams may find that such information useful when analyzing or describing the health system context that serves as the backdrop for RMNCH programs.

Public Expenditure Reviews (PER)

PERs, which the World Bank has been assisting low and middle income country governments conduct since the 1980s, are comprehensive analyses of public spending to examine a range of issues related to the allocation of public resources including the efficiency, equity, and sustainability of spending. Typically, PERs provide an overview of the country's macroeconomic and fiscal performance as well as an analysis of the composition of public spending (e.g., the proportion of recurrent versus capital spending and the allocation of the national budget across different sectors). Additionally, most PERs also offer in-depth analyses of spending in priority social sectors, with specific chapters dedicated to discussing public spending in sectors like health and education. Some countries have produced specialized PERs that examine a single sector. The 2006 Uganda Health PER is an example of a stand-alone PER for the health sector.

While there is no common methodology or framework for PERs, the exercises typically investigate the aggregate level of public sector spending and deficit, and its consistency with the country's macroeconomic outlook; spending across sectors and within sectors to assess alignment of spending with the goal of maximizing social welfare; the role of private versus public sector in financing and provision of social programs; the impact of key public programs for the poor, etc. (Pradhan, 1996). PERs can potentially help Countdown case study teams understand some of the macroeconomic and political issues that have influenced domestic and international resources available for RMNCH programs.

FURTHER READINGS AND RESOURCE MATERIALS

Powell Jackson et al., (2006) provide a detailed explanation of how CRS data can be used to track ODA for RMNCH. SHA 2011 is the definitive guide to NHA and provides definitions and explanations for all key terms and classifications (OECD et al., 2011). Teams can access country PETS and QSDS reports from World Bank's PETS/QSDS portal at http://pets.prognoz.com/prod/. The World Bank (2009) guide to producing PERs is a good resource for those interested in learning more about this HRT tool.

3. Financial Protection and Access to Services

The need of families to pay out-of-pocket (OOP) to access maternal and child health (MCH) services can be a significant barrier to improving MCH outcomes. Financial costs do not only consist of payments to obtain medical care, but also include expenses incurred in transportation and other economic costs such as loss of wages by being unable to work. For mothers and children seeking care at public facilities, costs are rarely dominated by official user fees, and can include the expense of having to purchase medicines and supplies that are not in stock, as well as informal payments to health care staff.

Such spending can deter use of needed services, and frequently financially impoverish families. A high level of costs is particularly common for mothers needing facility-based delivery, and the high expense of such care is often a major reason for low levels of skilled birth attendance and high maternal mortality. Financial barriers can play a leading role in explaining substantial socioeconomic inequalities in access to needed MCH services, and addressing such barriers is often necessary for reducing such disparities. In many countries, policies and interventions that have reduced the OOP costs of accessing MCH services have led to increased use of these services and improvements in MCH outcomes. To the extent that OOP costs can impoverish families or prevent them from escaping poverty, problems of financial protection also have a broader relevance to overall poverty reduction and MDG 1 to halve the proportion of people whose income is less than $1.25 a day by 2015.

From the perspective of Countdown's focus on why and how countries have made progress towards MDGs 4 and 5, analysts should consider the role that changes in financial barriers may have played in improving coverage of services and reducing inequalities in access, and the extent to which they hold back better outcomes. When financial barriers affect access, it is also necessary to assess their contribution relative to other factors, since these may actually be more important and easier to address. Finally, regardless of actual access, the direct impact on household budgets and well-being of having to pay for needed services should always be kept in mind.

Background

The term financial protection or financial risk protection refers to the extent to which patients or their households do not have to incur OOP expenditures (OOPE) that cause them hardship in order to access care. Large OOPE can leave households with so few resources that their standard of living falls below the poverty line. This is commonly described as impoverishment, and economists have developed a range of metrics to measure the extent and prevalence of this. Most of these involve counting the number of people whose net spending after accounting for health expenses falls below some poverty threshold, such as the one or two dollar international poverty lines. A related, but different notion of hardship refers to when a household has to spend such large amounts on obtaining health care that it appreciably reduces its overall wealth and assets. This is termed catastrophic expenditure, and economists typically measure this by counting the number of people who have to allocate more than a specific fraction of their overall spending to health expenses. More detailed discussion of these concepts and standard methods of measurement can be found in O'Donnell et al. (2008). Financial protection can defined as

the extent to which households can access needed care without experiencing significant levels of either catastrophic or impoverishing spending.

Possible Research Questions

Country case study teams should consider and try to address any or all of the following questions, asking always how these might affect overall use of MCH services and eventual MCH outcomes.

1. How much do households have to spend, both as direct medical expenses and also as other indirect costs, when obtaining MCH services? What do these expenses consist of and how do they vary between different groups, e.g., urban versus rural, or by income level?
2. To what extent does spending on MCH services have impoverishing or catastrophic impacts on families? What contribution does MCH OOPE make to overall health impoverishing and catastrophic expenditures?
3. How important is the cost of care in discouraging families from obtaining health care for mothers and children? How important in comparison to other factors such as lack of physical access to health facilities, poor quality of services, lack of health literacy and appreciation of the need for care, and other social factors? How does this vary between different groups in the population?
4. How have these changed over time? Do such changes help explain changes in overall coverage by and use of MCH services?

Potential Data Sources

Household surveys that have collected information about health care use and/or health expenditures are the primary source for examining these issues. In practice, no single survey will be suitable for investigating all these questions: comprehensive analyses will need to make use of multiple surveys. Countdown case study teams should do a full inventory of what surveys are available, checking whether they can obtain access and giving preference to nationally representative surveys. Surveys generally fall into two types:

1. Household budget surveys: These surveys are typically carried out by national statistical offices to assess household consumption and living standards or as a basis for tracking prices. In most countries, such surveys are conducted every few years. These surveys are best for analyzing overall health spending, and for measuring the impoverishing and catastrophic impacts of health OOPE. However, the standard budget survey is not so useful for analyzing MCH spending, since spending is usually recorded at the level of a household, and the linkage between spending and health care use or with specific individuals in a household is not recorded, which makes it impossible to separate out MCH from other health spending. Nevertheless, in most countries financial burdens of accessing MCH care differ little from those in accessing all care, and so general analyses can still be informative about the financial costs of receiving MCH care.

2. Household health and health care use surveys: Health ministries and agencies typically commission these types of surveys. The most common design asks whether individuals were sick, whether they sought treatment, and how much they spent, with information being recorded at the level of

individuals. Different surveys can add additional questions, such as the reasons why a sick person did not seek treatment, why these chose a particular provider, and the type of treatment obtained. These surveys are most useful for analyzing the composition of OOPE, which providers are used and why. DHS surveys are an example of a health and health care use survey, and are particularly suited for analysis of MCH utilization. However, the DHS does not generally support analysis of financial costs and barriers. The LSMS surveys are similar to a household budget survey, but usually add modules on illness and health care use, so providing elements of both types of survey.

Investigators should be aware of a number of limitations and design issues that can arise with all these surveys:

1. Length of reference period: Standard analyses of financial protection use surveys with one month recall of household spending. Surveys with significantly longer or shorter recall periods can be used to examine the distribution of financial risk within a population, but cannot be used to compare levels of financial protection between countries, because financial risk measures are sensitive to the reference period used.
2. Sample size: Most surveys do not contain an adequate sample to analyze maternal health care use and spending. Maternal events are much rarer than health care use and spending by children, and typically surveys do not have sufficient events to base reliable estimates. In these cases, analysis of financial barriers will usually be restricted to child health. The main exception to this is when surveys contain modules that specifically ask about maternal health care use and apply long recall periods, such as one year or more.
3. Socioeconomic status (SES) level: Analyses of disparities between rich and poor households depends on the survey having adequate measures of relative living standards. In general, the best measures for this require data on total household expenditure or consumption, followed by data on household assets, and then data on household income. If using expenditure or consumption, relative living standards should be assessed in terms of expenditure/consumption per adult equivalent, which takes into account household size and economies of scale. Health surveys tend not to have good data on household consumption/expenditure/income, but information on assets can be used to differentiate poor from non-poor households using principle component analysis (O'Donnell et al., 2008; Filmer and Pritchett, 2001).
4. Reason for obtaining care or cause of illness: The way in which surveys record these reasons varies and tends to be highly idiosyncratic. In most surveys, the pre-coded response categories are neither comprehensive nor adequate, and cannot be reliably used to identify maternal causes, unless this was specifically asked about. Consequently, these responses are often of limited value.
5. Comparability of survey findings between surveys: When multiple surveys are available, it can be useful to compare trends over time. However, comparisons are most meaningful when done using different rounds of the same survey, as differences in survey design (sampling, reference period, question design) can introduce significant biases into survey findings and render comparisons unreliable. In some cases, it is possible to control for differences in survey design, but not always.

Methods

Countdown case study teams can undertake the following types of analyses depending on the country context as well as the data that is available:

1. Use of health care when sick: If surveys ask about illness incidence and relate that to health care use, the team can examine the overall variation in reported sickness by socioeconomic status, and specifically for mothers and for children where possible. Although poor people should normally have worse health and MCH outcomes, it is normal to observe that poor people report less sickness than the non-poor. The size of this disparity can be an important point to the existence of large barriers to access which condition people to think they don't need care, as well as the importance of differences in health awareness and literacy as explanations for inequalities in health care use. The percentage of those who report they are sick who go on to obtain medical care should be analyzed, and can be a separate indicator of disparities in access to health care.

2. Levels, composition and distribution of MCH OOPE: Any survey that records OOPE by type of expenditure is adequate for examining the composition of OOPE and its distribution across the population. If expenditure is recorded in combination with the type of care and provider, it can be useful to examine the variation in average OOP cost by the same, as well the composition of the spending. This may shed light on why particular providers are chosen, and often reveals that nominally free public services are associated with substantial costs in use. When nominally-free public services are not actually free, it is important to look at what costs public sector patients incur and why: e.g., are costs due to official charges, informal payments or because of inadequate supplies. In many cases, it is useful also to look at the relative role of medical fees, medicines and hospital charges in OOPE.

3. Impoverishing and catastrophic expenditures: The most commonly used methods and computational procedures are described in detail by O'Donnell et al. (2008). For catastrophic impact measurement, we would recommend using as indicator the percentage of people whose households spend more than 10%, 20% and 40% of their non-food expenditure on medical care. For impoverishment, we would recommend looking at the number of people forced below the "two dollar" World Bank poverty line. A general analysis of the incidence of these expenditures can provide important information about the overall level of financial protection in the health system. Depending on data availability, this can be extended by looking at which items of spending contribute most, e.g., medicines outpatient doctor fees, inpatient costs, etc., and what contribution OOPE for MCH makes to the overall impact.

4. Role of financial and other barriers in reducing access to care: If significant disparities in use of health care by sick individuals are observed, two analytical strategies are possible. The easiest is if a survey asks why people did not seek care. Here one should attempt to categorize possible reasons into those of financial cost, distance, quality of care, and health awareness or if the individual thought it was necessary to obtain care, and one should check if these reasons vary by SES and by urban and rural residence. Typically, financial cost and distance are most important barriers for poor

mothers and children, whilst quality of care concerns will be more important for the non-poor. The second and alternative analytical approach is to use multivariate modeling to examine the decision to seek care when sick, and the choice of providers. This requires the ability to estimate health care choice and demand models. One common limitation of these models is the difficulty of factoring in the impact of variations in the distance to, price and quality of provider different providers, although this can be managed by using methods such as hedonic pricing. However, these methods are only recommended for those with appropriate econometric expertise, as they are analytically demanding.

The choice and use of household surveys in Afghanistan case study

Afghanistan lacks a tradition of regular surveys, and many surveys are one-off activities. It does, however, have a household living standards survey, the National Risk and Vulnerability Assessment (NRVA) survey conducted in 2005 and 2007, and a health survey (Afghanistan Health Survey, AHS) conducted in 2006 and 2012. The NRVA was used to analyze patterns and changes in spending, and the impoverishing and catastrophic impacts of OOPE, with SES being measured using household expenditure per adult equivalent. It could not be used to analyze health care use, for which the AHS was used. However, the AHS did not collect data on household expenditure, so the SES measure used in the NRVA could not be applied to the AHS data. This problem was circumvented by using an asset index to rank the AHS households, and by tabulating results from the NRVA and AHS by SES quintiles to allow comparisons. Owing to the security situation, neither of the two AHS surveys could cover all parts of the country, and each covered different districts, making direct comparisons of changes over time erroneous. To allow comparisons of changes over time, a subset of the main analysis was done using data only from those districts that were surveyed in both rounds.

These analyses can shed considerable light on the role of financial costs and barriers to care. However, by themselves they are usually not sufficient to explain why MCH outcomes improved in a case study country. To do that, these findings must be combined with information on changes in health care use and health outcomes, as well as changes in the overall financing situation. For example, in Afghanistan the analysis of household survey data found that health care use had increased and was associated with a fall in out OOPE by public sector patients. Analysis of other administrative data confirmed the increase in health care use, and also showed a sustained increase in availability of medicines at all facilities, confirming this is as the most likely explanation for the fall in OOPE costs and potentially a key contributor to the increase use of all MCH services. See box for more information about the Afghanistan case study.

FURTHER READINGS AND RESOURCE MATERIAL

Brearley et al. (2012) provides a useful literature review of work on the household impact of OOPE for MCH that may be useful in providing a broader perspective for individual country findings, and for thinking about potential issues. Almost all of the suggested analyses require the ability to clean and analyze household survey data using statistical software. For details of how to conduct specific analyses

of financial protection and inequalities in health care use, the best guide is the World Bank publication on analyzing health equity with household survey data (O'Donnell et al., 2008). This provides not only extensive discussion of most analytic methods, but also Stata programming code for those inclined. For several practical examples of these analytical methods being used to examine MCH health care use, a good source is a series of analyses of data from several Asian countries published by the Asian Development Bank (Anuranga et al., 2012a, b; Chandrasiri et al., 2012; Jayanthan et al., 2012; Rannan-Eliya et al., 2012), in addition to the detailed analysis done in the Countdown Afghanistan case study (Wijemanne et al., 2014).

4. Equity Analysis

Background

Benefit incidence studies have a long history, but interest in this subject only began to surge in the 1970s with Robert McNamara's confidence that government spending could potentially alter income distribution and living standards of the poor in developing countries (Selden and Wasylenko, 1992). In other words, public spending should promote equity by improving economic welfare, while correcting for market failures (McIntyre and Ataguba, 2011). Benefit incidence analysis (BIA) considers which socio-economic group is receiving what benefits from using publicly subsidized government programs. BIA is defined as "a method of computing the distribution of public expenditure across different demographic groups, such as women and men…[which] involves allocating per unit public subsidies (for example, expenditure per student for the education sector) according to individual utilization rates of public services" (Alexander and Baden, 2000).

Such analyses have demonstrated that health care spending in parts of Africa is not well targeted to the poorest populations. Castro-Leal et al. (1999) used BIA to demonstrate that in Cote d'Ivoire, Ghana, Guinea, Kenya, Madagascar, South Africa, and Tanzania the share of total subsidies to the poorest quintile was significantly less compared to the richest 20%. In contrast, studies in several countries with good performance in maternal mortality reduction, such as Jamaica, Malaysia and Sri Lanka, have consistently found pro-poor targeting of government health spending. In low and middle-income countries, the World Bank has conducted most of the BIA studies focusing on publicly funded health and education services and distribution of benefits. For education spending in South Africa, Indonesia, Bolivia, and Paraguay it was found that public spending on education benefit the poor and in some instances this benefit is only with lower (primary) public education spending (Castro-Leal 1996; Lanjouw et al., 2001; Ajwad and Wodon, 2002). However, education spending in Côte d'Ivoire, Ghana, Guinea, Kenya, Madagascar, South Africa, and Tanzania favors the non-poor (Castro-Leal et al., 1997; Demery et al., 1995). For Yemen safety net and poverty programs were analyzed and found low program coverage and poor targeting among these programs (van de Walle, 2002). For heath spending, Indonesia's public spending is pro-poor for primary health care, while in Ghana public spending on health is hardly reaching the poor (Lanjouw et al., 2001; Demery et al., 1995).

Note, this analysis cannot reflect the variation in the quality of services provided to different groups of users and therefore BIA assumes that quality levels is consistent across all areas in a country (IMF, 2003). Many BIA analyses also do not adequately account for variations in spending levels across different regions in a country, assuming that similar services are resourced equally in all locations.

A BIA looks at equity in a different way compared to the equity component for the Countdown case study. The equity component assesses key RMNCH coverage and outcome indicators such as contraceptive acceptance rate, antenatal care, immunization coverage, and mortality across different wealth quintiles. BIA assesses whether public spending is targeted to the poorest populations.

Possible Research Questions

Countdown case study teams could use BIA to explore the following questions:

1. Is public spending on RMNCH significantly more for the bottom 20% and 40% of population compared to the richest 20%?
2. How does this relate to the findings from the equity analysis?

Potential Data Sources

Conducting a BIA requires data on public spending on a service (net of any cost recovery fees, out of pocket expenses by users of the service, or user fees) and utilization, a socio-economic status measurement, and unit cost data (meaning the cost for each type of health service per individual/socio-economic group) of different types of health services. Government spending data are typically obtained from budget execution data as reported by the ministry of finance, the relevant line ministry, or the central statistical agency (IMF, 2003). Data on utilization, socioeconomic status, and unit cost can be obtained from a household survey or health information system. A BIA cannot be conducted without the knowledge of utilization and socioeconomic status of the population using the services.

Methods

BIA involves a seven-step process that can be easily implemented using Excel (IMF, 2003; McIntyre and Ataguba, 2011):

1. Select a measure of living standard or socio-economic status (SES) and rank the populations of users from poorest to richest using identified welfare measure, and aggregate them into groups with equal numbers of users.
    a. The unit of analysis in a household survey can either be the household, comprising all family members living together or an individual within the household. The welfare measure is typically either income or consumption. Both the household survey information and welfare measure are needed to rank users.
    b. The choice of welfare measure for ranking users (from poorest to richest) can also make a significant difference to estimates of benefit incidence. The most widely used indicator is per capita household expenditure, in which each member of the household receives an equal weight. An alternative indicator is per adult equivalent household expenditure, which takes into account the higher consumption needs (welfare) of the adults; as a result, adults are given a higher weight than children no consistent pattern or no general rule seems to exist in this area. Therefore, it makes sense to report benefit incidence based on both welfare measures. Many BIA studies tend to report only the per capita measure.
2. Estimate the utilization of different types of health services by individuals/different socio-economic groups (services such as primary level clinics, district hospitals, regional hospitals and central hospitals for public sector services[3]).
3. Calculate the average unit cost of providing a public service by dividing government spending on

[3] It is possible to conduct such an analysis for private health care services as well. In which case, categories such as general practitioners, specialists, retail pharmacies and private hospitals would have to be included in this step.

the service (net of any cost-recovery fees and out-of-pocket expenses by the users) by the total number of users of the service (ultimate beneficiaries of the service - e.g., students enrolled in primary schools or patients visiting a health center).

4. Multiply utilization rates by unit costs for each type of health service for each individual/socio-economic group.
5. Aggregate benefits of utilization (or of public subsidies), expressed in monetary terms, across different types of health service for each individual/socio-economic group.
    a. This step implicitly assumes that the average benefit from or cost of a service delivery does not vary with income or consumption level, or indeed any other factor. BIA typically assumes that the quality of a service is the same in rural and urban areas and ignores the potential effects of corruption or uneven quality of the public expenditure management, both of which can produce different benefits to users.
6. Compare the distribution of health service benefits (or of public subsidies) to some target distribution (e.g., relative to need for health care).
    a. From a policy point of view, this last step is the most important component of a BIA since it informs policymakers on how well government spending on a service is targeted, how it compares with the incidence of other types of government spending (e.g., primary health care vs. secondary and tertiary health care), or how the resulting benefit incidence stacks up against the past incidence of government spending in the same country or against incidence of spending in other countries.

It is possible to combine already established tools with a supplemental survey, such as using NHA expenditure data[4] with utilization data from health information systems (HIS). If a HIS is not well maintained, then it is possible to supplement the NHA data with utilization data obtained from a household survey and obtain data on any outpatient and inpatient services utilized, as well as information on all visits – visits to either the same provider or to more than one service provider (McIntyre and Ataguba, 2011). A BIA conducted by McIntyre and Ataguba (2011) in South Africa used a district HIS and obtained information on utilization of private sector services from the private health insurance organizations since these services are primarily used with private insurance coverage. Given these detailed criteria, surveys such as the Demographic Health Surveys (DHS) or Living Standards Measurement Surveys (LSMS) are not adequate for a comprehensive and accurate BIA.[5]

Two ways that benefit incidence data can be presented is either by a simple percentage share of total benefits for each quintile or by concentration curves and indices (McIntyre and Ataguba, 2011). A concentration curve "plots the cumulative percentage of the health variable (y-axis) against the

[4] If NHA data is not available then health expenditure data could be obtained from the Ministry of Health.

[5] According to McIntyre and Ataguba (2011), LSMS collects information on utilization of health services, but generally does not allow for accurate calculation of utilization rates for specific types of services and providers; while the DHS collects this type of data more broadly such as if a child sought treatment for a fever and where (health facility or traditional healer) or whether a mother received any ante- or post-natal care and where, it is limited in scope to specific maternal and child indicators and does not account for frequency and specific services used.

cumulative percentage of the sample, ranked by living standards, beginning with the poorest, and ending with the richest (x-axis)" (World Bank, 2014a). While the concentration index quantifies the degree of socioeconomic-related inequality in a health variable and is directly related to the concentration curve – a positive value for the index means pro-rich and a negative value means pro-poor (World Bank, 2014b).

Figure 4.1 illustrates the distribution of benefits by wealth quintile from outpatient visits in public sector facilities in South Africa. In this instance, it is difficult to see whether or not a particular service is pro-poor. In this case, one needs to look at the concentration index to determine whether the benefits are pro-poor or pro-rich. The sign of the concentration index indicates whether public spending is pro-rich or pro-poor: If the concentration index is negative then public spending is pro-poor; if this index is positive then public spending is pro-rich; and if the index is 0 then there is no inequality in public spending. Here, the distribution of public outpatient benefits in South Africa is considered "pro-rich" (albeit marginally) given that the concentration index is 0.0046 (McIntyre and Ataguba, 2011).

However, it is possible that the concentration index is positive, signifying pro-rich distribution of benefits, when the concentration curve may demonstrate that this is the case only for certain socio-economic groups. Using the same McIntyre and Ataguba (2011) South African example, the concentration curve in Figure 4.2 illustrates that the distribution of regional hospital inpatient care is pro-rich among the lower quintiles but pro-poor among the upper quintiles. With these two effects, the regional hospital inpatient care is considered to be overall pro-rich with a concentration index of 0.073 (McIntyre and Ataguba, 2011).

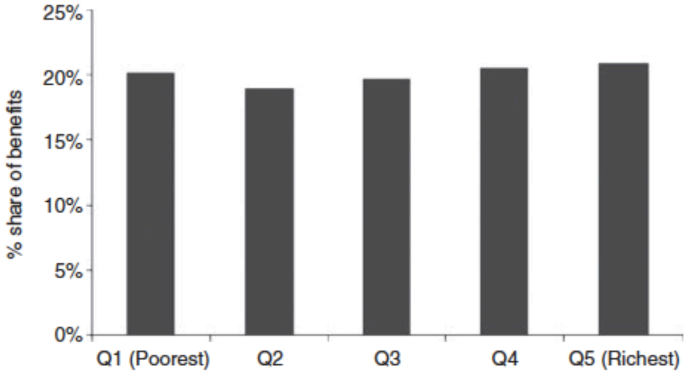

Figure 4.1: Percent share of public outpatient benefits (Source: McIntyre and Ataguba, 2011)

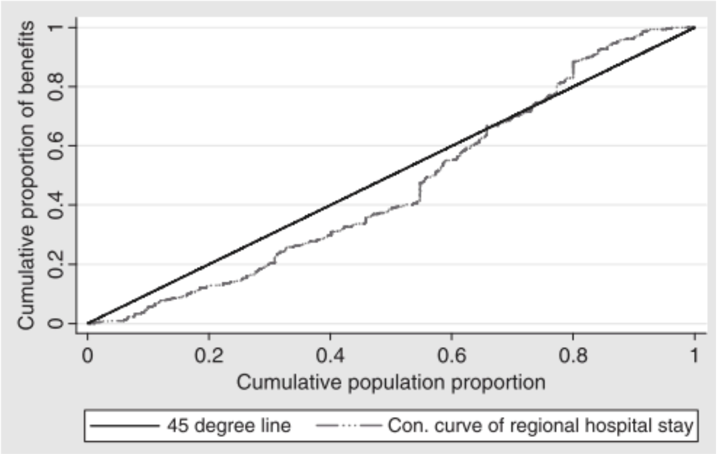

Figure 4.2: Concentration curve of benefits from regional hospital inpatient care (Source: McIntyre and Ataguba, 2011)

For more information on calculating concentration curves and indices refer to the following link: http://www.worldbank.org/en/topic/health/publication/analyzing-health-equity-using-household-survey-data.

Limitations

BIA could be an effective approach to understanding who is benefiting the most from public health spending. However, despite its usefulness, there are a few limitations to this analysis. BIA is just one piece of the puzzle in understanding the broader scope of resource tracking, and findings need careful interpretation and explanation of how the BIA was conducted and data used (Pearson, 2002). Unit cost data could be a potential limitation. First, it might not account for user fee payments made by the patient for publicly funded services (Wagstaff, 2010), unless these are netted out. Secondly, if budget allocation data are the only available source compared to actual recurrent expenditures, this limits interpretation of the BIA. Third, this analysis makes a strong assumption that cost data is a good proxy for benefit that users receive from government services (IMF, 2003). Mahal, Yazbeck, and Peters (2001) make the points that equity in use of resources and services is not necessarily the same as impact; BIA analysis does not account for quality of care; and cannot measure benefits to society from services with positive externalities (e.g., immunizations). Additionally, variations in costs of services could be masked when there is little disaggregation by facility type, types of services, or region of the country (Castro-Leal et al., 1999). As Pearson (2002) stated, the major constraints for obtaining such disaggregated data are cost and feasibility.

FURTHER READINGS AND RESOURCE MATERIAL

McIntyre and Ataguba (2011) offer a comprehensive guide to undertaking BIA. The World Bank has developed user-friendly software for undertaking equity analysis called ADePT Health, which can be accessed from: http://econ.worldbank.org/WBSITE/EXTERNAL/EXTDEC/EXTRESEARCH/EXTPROGRAMS/EXTADEPT/0,,contentMDK:22681679~menuPK:7108377~pagePK:64168182~piPK:64168060~theSitePK:7108360,00.html. There are

numerous country applications of BIA for public social spending that can serve as a reference for country case study teams, such as Mahal et al., (2000) for India and Mogues et al., (2011) for Ethiopia. Please visit http://web.worldbank.org/WBSITE/EXTERNAL/TOPICS/EXTPOVERTY/EXTPSIA/0,,contentMDK:20472485~menuPK:1108016~pagePK:148956~piPK:216618~theSitePK:490130~isCURL:Y~isCURL:Y~isCURL:Y,00.html for additional documents on BIA.

5. Costing

Costing models estimate the resources required to undertake actions to achieve public health goals. The Partnership for Maternal, Newborn and Child Health commissioned a technical review of costing tools (Bitran y Asociados and PATH, 2008). Most of the information in this section is drawn from this report.

Background

Countries use costing tools for different purposes, such as in their planning and budgeting processes to measure the resources needed for particular plans or programs, for allocating resources to facilities, for setting user fees, etc. Most existing costing models are focused on specific diseases or health areas typically corresponding with the MDGs. The models assess the costs of implementing interventions related to the focus disease or health area to achieve particular coverage targets or health outcomes. The user typically specifies a target coverage level or a specific health outcome, and the models calculate the cost of achieving that goal taking into account various demographic considerations, the quantity of inputs needed, the prices of inputs, the effectiveness of health interventions, etc. The technical review report (2008) provides the following example to illustrate the logic underlying costing models:

> ..costing a child health intervention such as Vitamin A supplementation would first involve identifying inputs needed to deliver the intervention, such as the 100,000 and 200,000 IU [International Units] Vitamin A capsules, the community health workers to distribute them and the community awareness campaigns to be run to educate the population about the upcoming health fair at which children will receive the supplementation. The way in which these inputs are combined to produce the intervention is the intervention production function. The intervention cost is calculated by multiplying the unit price of the two types of capsules times the number of capsules to be distributed, plus the number of health workers needed multiplied by the number of days they work multiplied by their daily pay rate, plus the cost of each community awareness campaign multiplied by the number of campaigns. From this point an intervention cost can be calculated and the budgetary impact and financing implications analyzed.
>
> To determine the health outcome of the child health intervention, the health production function calculates the impact of Vitamin A supplementation on child health status under the assumption that the supplementation is 75% effective in reducing under-5 mortality in underweight infants. The coverage level can then be determined by calculating what percentage of children aged 6-11 months will be reached with this intervention. This intervention can be delivered as a one month, one year or multiple year activity. Planners must also take into consideration the impact of the country's macroeconomic conditions that affect the health system performance. For example, slow economic growth decreases available resources for health and other sectors, and low incomes that are linked to malnutrition increase the need of Vitamin A supplementation.

There are over a dozen costing models related to the MDGs developed by different agencies and institutions. Table 1 below highlights some that are relevant for studying the costs of RMNCH-related interventions.

Tool	Developers	Focus Interventions	Countries that had used the tool (as of 2008)
Marginal Budgeting for Bottlenecks Toolkit	UNICEF and World Bank	Child and adult immunizations; child health interventions; family planning; general health systems improvements; HIV/AIDS prevention and treatment; malaria prevention and treatment; maternal health interventions; TB prevention and treatment	Angola, Benin, Burundi, Burkina Faso, Cameroon, Comoros, Cote d'Ivoire, Ethiopia, Ghana, Guinea, Guinea Bissau, India, Lesotho, Liberia, Madagascar, Malawi, Mali, Mauritania, Mozambique, Niger, Nigeria, Rwanda, Sierra Leone, Swaziland, Uganda and Zambia
RH Costing Tool	UNFPA	Child health interventions (newborns only); family planning; general health systems improvements; HIV/AIDS prevention and treatment; malaria prevention and treatment (in the context of antenatal care); maternal health interventions	Armenia, Azerbaijan, Dominican Republic, Ethiopia, Georgia, Ghana, Indonesia, Lao PDR, Mongolia, Tajikistan, Turkey, Uganda, Yemen and Zimbabwe
The Integrated Healthcare Technology Package (iHTP)	WHO	Child and adult immunizations; child health interventions; Family planning; General health systems improvements (partially); HIV/AIDS prevention and treatment; Malaria prevention and treatment; Maternal health interventions; TB prevention and treatment	China, Democratic Republic of Congo, Kyrgyzstan, Malawi, Mexico, Mozambique, Namibia, South Africa, Sri Lanka and Ukraine
CORE Plus	Management Sciences for Health	Child and adult immunizations; Child health interventions; Family planning; General health systems improvements; HIV/AIDS prevention and treatment; Maternal health interventions; TB prevention	Haiti, Rwanda, Afghanistan, Bangladesh, Bolivia, Ethiopia, Guatemala, Haiti, Honduras, Kenya, Madagascar, Mexico, Nicaragua, Rwanda, Senegal, South Africa, Tanzania, the

		and treatment	United States and Zimbabwe
Integrated Health Model	UNDP	Child and adult immunizations; Child health interventions; Family planning; General health systems improvements; HIV/AIDS prevention and treatment; Maternal health interventions; TB prevention and treatment	Haiti, Nigeria, Rwanda and Uganda
Child Health Cost Estimation Tool	WHO	Child Health Interventions; Malaria prevention and treatment	Cambodia, Mozambique and Uganda

In response to requests from countries to harmonize the various disease-specific costing models, many of the agencies that developed existing models came together to form the UN Inter Agency Working Group on Costing, which released the OneHealth Tool in May 2012. This tool links the disease-specific components of the other costing models to provide a health systems perspective. According to its authors, the tool offers policy-makers and planners a single framework for planning, costing, and analyzing the impact of strategies for all major diseases and health system components. The tool has been implemented in more than 25 countries, mostly in Sub-Saharan Africa.

Application to Country Case Studies

Undertaking fresh costing analysis is in all likelihood beyond the scope of the Countdown case studies, as it requires a considerable investment in terms of resources for training and time for training, data collection and analysis. However, case study teams could benefit from any existing RMNCH-related costing studies that have been done. Such exercises might provide valuable information about the resources necessary for scaling up RMNCH-related interventions. By juxtaposing this information with estimates of resources available for RMNCH program (e.g., from NHAs), country case study teams could investigate the adequacy of investments for RMNCH programs. It is also worth noting that for many countries the OneHealth tool comes preloaded with data. The team is encouraged to investigate how much data is available in the OneHealth tool and the source for the information before using the tool.

Further Readings and Resource Material

The Final Reports of Technical Review of Costing Tools (Bitran y Asociados and PATH, 2008) provides a good introduction to costing and a comprehensive overview of existing costing tools. Case study teams can access more information about the OneHealth Tool at http://www.futuresinstitute.org/onehealth.aspx.

6. Country Examples

This next section showcases how the financial component was conducted for the Countdown case studies in Peru and Ethiopia, providing further examples for the case study teams on what could be feasibly done in different contexts.

6.1. Peru

Peru is among the countries with the highest burden of maternal and child deaths. Changes in socioeconomic standards and sanitary conditions in the last decade have led to a remarkable decrease in the number of such deaths, as well as improvements in a variety of indicators linked to RMNCH. The aim of the case study was to identify the key elements that explain these improvements and draw out lessons that can be shared with other countries in the world.

As the first step, the case study team developed a conceptual framework to highlight the role of factors expected to be linked to the RMNCH improvements, such as (a) changes in social, political and economic determinants of health; b) changes in sectors closely linked to the health system, such as water and sanitation, education, cash transfers, and women's empowerment; c) changes in health system policies, financing and programs; d) improvements in access, quality and equity of RMNCH interventions, and e) impact on improved health, survival and nutrition of mothers and children. Based on the conceptual framework, the team undertook both quantitative and qualitative analyses. The qualitative component included discussions with key experts in the field regarding the main policies, programs and factors linked to RMNCH and their potential impact. The quantitative analysis focused on identifying the factors that explained changes in the impact variables. Specifically, the team performed multilevel mixed-effects linear regression analysis on outcome measures related to child health as well as maternal and neonatal health.

Although Peru has an advanced health information system, its coverage of financial data has limitations. Financial data that is available is incomplete and presented in varying formats, making it hard to analyze. The first challenge faced by the team was to integrate the data covering the various RMNCH components, both at national and subnational level. The country recently released a NHA report with health expenditure estimates for the period 1995-2012, which was useful to understand the environment where RMNCH financing is developed. According to the health accounts, there are mainly three financing entities in Peru: households, government and social security. In the last decade, household spending reduced while utilization of services increased as a result of improved access, especially for households with low income. It is likely that RMNCH services benefitted from this situation. It was not possible to obtain or generate a household RMNCH component. A key question that remains is how different is the financing profile for RMNCH from the one for total health.

In Peru, the government has developed a clear policy to improve health during the last decade, with health spending increasing faster than GDP. Government spending, which includes spending by both the Ministry of Health and the *Seguro Integral de Salud,* represents around 30% of RMNCH spending. It has increased to the point where it is at par with per capita spending through health insurance. This has

modified drastically the structure of spending in RMNCH. However, political support for reproductive health has fluctuated considerably, as a result of which their financing has been more unstable than other items of health spending. The current policy of enhancing the right to health has ensured the financing to the RMNCH components, which is now provided through a results-based budgeting modality. Additionally, a fund to cover specific population groups was created, which has progressively increased coverage and improved financing in specific areas and population groups. This fund is growing to offer wider benefits. The Peruvian government is in search of more efficient ways to finance health services, including those related to RMNCH. It is expected that major improvements will reach some of the subnational areas through which coverage can be improved further.

External financing for RMNCH is minimal. While the level of funding for all channels is decreasing, private grants are becoming relatively more important. Also, development partners play an important role beyond the level of funding they provide because of the catalytic effect they have had on RMNCH programs.

This financing analysis has been useful to understand Peru's progress towards improving RMNCH outcomes. However, it is a work in progress. The country is moving to produce yearly SHA 2011 in which both the subnational level as well as the distribution by disease/program will be available and the RMNCH components may be made available. Comprehensive information on health spending that is routinely produced will allow analysts to better understand the ongoing changes in the country.

6.2. Ethiopia

The focus of the case study is identifying the contributing factors that enabled Ethiopia to reach MDG 4 before the 2015 deadline. One important area that led to this success is the Government's ability to efficiently provide the resources needed to implement effective programs that greatly reduced under-5 child mortality. Ethiopia has made significant achievements to streamline Government and donor funds for health through its harmonization initiative, reduced OOPE from 1995 to 2011 according to the NHAs, and continue to implement financial programs and policies that are expected to reduce the burden of health care costs on households (such as the fee-waiver program).

The financial component of this case study attempts to explore if there is an association between trends in health expenditures and key health policies and programs, and how trends in health care expenditures have influenced RMCH coverage and outcome indicators. These research questions are linked with the other components of the case study (RMCH health system policy, coverage, and equity analysis) in order to provide a comprehensive picture of how Ethiopia achieved MDG 4 prior to 2015.

In order to answer the research questions outlined above, the case study team used secondary data sources that were politically accepted. This consisted of extracting data from five rounds of NHAs and a review of other relevant documents such as Ethiopia's Health Care and Financing Strategy (1998). Microsoft Excel was used to make tables and graphs to demonstrate trends in total, reproductive, and

child health expenditures between 1995-2011. These tables and graphs were then compared to the health system policy analysis and coverage findings.

The analysis found that a rapid increase in Ethiopia's total health expenditure, especially between 2004/2005 and 2010/2011, is linked to the rapid scale-up of the Health Extension Program (HEP) as well as service delivery expansion through program implementation like community based nutrition program (CBN) and community management of acute malnutrition programs (CMAM). Additionally, Ethiopia was able to rapidly mobilize resources from external sources particularly after the Ethio-Eritrean war (ended in 2001), due to high political commitment on the health sector by the Government of Ethiopia. Between 1995-2005, the total health expenditure (THE) in US dollars (USD) increased by 127% while between 2005-2010 – after the rapid expansion of HEP and other health programs – THE increased by 215%. THE per capita in 1995/1996 was USD 4.09, which increased dramatically by 2010/2011 to USD 20.77. Although this is not achieving the minimum benchmark set by the World Health Organization of USD 34 per capita, Ethiopia is on the right track. Total child health expenditures increased more rapidly between 2007/2008 and 2010/2011 with a percent change of 179%, compared to only 22% between 2004/2005 and 2007/2008. The decrease in under-5 child mortality rate (from 168 per 100,000 live births in 2000 to 88 in 2011) corresponds with the increase in child health expenditures (101.32 million birr in 2004/2005 to 185.5 million birr in 2010/2011) over time. Furthermore, the percent decline in the under-5 child mortality rate explained by the Lives Saved Tool (LiST) for the periods 2000-2005 and 2005-2011 is 25% and 60%, respectively. LiST is a computer projection model used to estimate the number of deaths that can be averted as a result of scaling up effective maternal and child health interventions. This suggests that the implementation of new and effective programs like HEP, National Nutrition Program, and scale-up of other effective interventions post-2005 have made a difference in reducing under-five mortality. These programs and interventions were supported by increased health care financing as reflected in the reproductive and child health accounts in 2005-2011 (185.5 million birr in 2010/2011) compared to 2000-2005 (101.32 million birr).

The findings from policy, service coverage, equity and LiST analyses revealed that rapid change in most of the components of the case study were observed after 2005 – a turning point of improving maternal and child health for Ethiopia. Not only is this attributed to the development of key policies and strategies but also the financial commitment to effectively implement them.

References

Aiddata. n.d. www.aiddata.org. Accessed on November 5, 2014.

Ajwad, M and Wodon, Q. 2002. Who Benefits from Increased Access to Public Services at the Local Level? A Marginal Benefit Incidence Analysis for Education and Basic Infrastructure. World Bank Economists' Forum Vol. 2 (2002), pp. 155–175. http://siteresources.worldbank.org/INTPSIA/Resources/490023-1121114603600/13916_part3b.pdf

Alexander, P., and S. Baden. 2000. Glossary on macroeconomics from a gender perspective, Report No 48. BRIDGE, Institute of Development Studies, University of Sussex, United Kingdom.

Anuranga, C., J. Chandrasiri, R. Wickramasinghe, and R.P. Rannan-Eliya. 2012b. The Impact of Out-of-Pocket Expenditures on Families and Barriers to Use of Maternal and Child Health Services in the Lao People's Democratic Republic: Evidence from the Lao Expenditure and Consumption Survey 2007–2008 RETA–6515 Country Brief. Manila: ADB.

Brearley, L., S. Mohamed, V. Eriyagama, R. Elwalagedara, and R.P. Rannan-Eliya. 2012. Impact of maternal and child health private expenditure on poverty and inequity: Review of the Literature on the Extent and Mechanisms by which Maternal, Newborn, and Child Healthcare Expenditures Exacerbate Poverty, with Focus on Evidence from Asia and the Pacific. Manila: ADB.

Castro-Leal, F. 1996. Poverty and Inequality in the Distribution of Public Education Spending in South Africa. Poverty and Social Policy discussion paper; no. PSP 102. Washington, D.C.: The World Bank. http://documents.worldbank.org/curated/en/1996/12/439092/poverty-inequality-distribution-public-education-spending-south-africa

Castro-Leal, F., Dayton, J., Demery, L., & Mehra, K. 1999. Public social spending in Africa: Do the poor benefit? The World Bank Research Observer. Issue 14.

Chandrasiri, J., C. Anuranga, R. Wickramasinghe, and R.P. Rannan-Eliya. 2012. The Impact of Out-of-Pocket Expenditures on Poverty and Inequalities in Use of Maternal and Child Health Services in Bangladesh: Evidence from the Household Income and Expenditure Surveys 2000– 2010 RETA–6515 Country Brief. Manila: ADB.

Chandrasiri, J., C. Anuranga, R. Wickramasinghe, and R.P. Rannan-Eliya. 2012. The Impact of Out-of-Pocket Expenditures On Families and Barriers to Use of Health Services In Pakistan: Evidence from the Pakistan Social and Living Standards Measurement Surveys 2005–07 - RETA– 6515 Country Brief. Manila: ADB.

Davoodi, H; Tiongson, E; and Asawanuchit, S. 2003. How Useful are Benefit Incidence Analyses of Public Education and Health Spending? International Monetary Fund (IMF) Working Paper. WP/03/227.

Demery, L; Chao, S; Bernier, R; Mehra, K. 1995. The incidence of social spending in ghana. Poverty and social Policy discussion paper; no. PSP 82. Washington, D.C.: The World Bank. http://documents.worldbank.org/curated/en/1995/11/440113/incidence-social-spending-ghana

Filmer, D., and L. Pritchett. 2001. "Estimating wealth effects without income data or expenditure data - or tears: Educational enrollment in India." Demography 38 (1):115–132.

Bitran y Asociados and PATH. 2008. Final Reports of Technical Review of Costing Tools.

Gauthier, B. and Z. Ahmed. 2012. “Public Expenditure Tracking Survey (PETS) and Quantitative Service Delivery Survey (QSDS) Guidebook.”

Hsu, J., C. Pitt, G. Greco, P. Berman, and A. Mills. "Countdown to 2015: changes in official development assistance to maternal, newborn, and child health in 2009–10, and assessment of progress since 2003." The Lancet 380, no. 9848 (2012): 1157-1168.

Jayanthan, J., W. Irava, C. Anuranga, and R.P. Rannan-Eliya. 2012. The Impact of Out-of-Pocket Expenditures on Families and Barriers to Use of Maternal and Child Health Services in Papua New Guinea: Evidence from the Papua New Guinea Household Survey 1996 and Household Income and Expenditure Survey 2009–2010 - RETA- 6515 Country Brief. Manila: ADB.

Kindornay, S., & Besada, H. 2011. Multilateral Development Cooperation: Current Trends and Future Prospects. Canadian Development Report 2011; Global Challenges: Multilateral Solutions, 37-52.

Lanjouw, P; Pradhan, M; Saadah, F; Sayed, H; Sparrow, R. 2001. Poverty, education, and health in Indonesia: who benefits from public spending? Policy, Research working paper series; no. WPS 2739. Washington, DC: World Bank. http://documents.worldbank.org/curated/en/2001/12/1660258/poverty-education-health-indonesia-benefits-public-spending

Mahal, A., Yazbeck, A. S., & Peters, D. H. 2001. The Poor and Health Service Use in India, (August).

Martinez Alvarez, M. 2014. “Countdown Case Study: Tanzania.” Presentation on at the Countdown Case Study Workshop in Nairobi, Kenya on February 27, 2014.

McIntyre, D., & Ataguba, J. E. 2011. How to do (or not to do) ... a benefit incidence analysis. Health policy and planning, 26(2), 174–82. doi:10.1093/heapol/czq031.

Nguyen, H., Snider, J., Ravishankar, N., & Magvanjav, O. 2011. Assessing public and private sector contributions in reproductive health financing and utilization for six sub-Saharan African countries. Reproductive health matters, 19(37), 62-74.

O’Donnell, O., E. van Doorslaer, A. Wagstaff, and M. Lindelow. 2008. Analyzing Health Equity Using Household Survey Data: A Guide to Techniques and Their Implementation, WBI Learning Resources Series. Washington, D.C., USA: World Bank.

OECD, Eurostat and WHO. 2011. System of Health Accounts. http://www.who.int/health-accounts/methodology/en/.

OECD. 2000. A System of Health Accounts, Version 1.0. http://www.oecd.org/health/health-systems/21160591.pdf.

OECD. Aid Statistics. http://www.oecd.org/dac/stats/. Accessed on November 1, 2014.

Patel, P., Roberts, B., Guy, S., Lee-Jones, L., & Conteh, L. 2009. Tracking official development assistance for reproductive health in conflict-affected countries. PLoS medicine, 6(6), e1000090.

Pearson, M. 2002. Benefit incidence analysis: how can it contribute to our understanding of health systems performance? DFID Health Systems Resource Centre (Vol. 44). London.

Pitt, C., Greco, G., Powell-Jackson, T., & Mills, A. 2010. Countdown to 2015: assessment of official development assistance to maternal, newborn, and child health, 2003–08. The Lancet, 376(9751), 1485-1496.

Powell-Jackson, T., & Mills, A. 2007. A review of health resource tracking in developing countries. Health Policy and Planning, 22(6), 353-362.

Powell-Jackson, T., Borghi, J., Mueller, D. H., Patouillard, E., & Mills, A. 2006. Countdown to 2015: tracking donor assistance to maternal, newborn, and child health. *The Lancet*, *368*(9541), 1077-1087.

Pradhan, S. 1996. “Evaluating Public Spending: A Framework for Public Expenditure Reviews” World Bank Discussion Paper 323. The World Bank: Washington, DC.

Rannan-Eliya, R. P., R. Hafez, C. Anuranga, and R. Wickramasinghe. 2012. The Impact of Out-of-Pocket Expenditures on Families and Barriers to Use of Maternal and Child Health Services in Timor-Leste: Evidence from the Timor-Leste Surveys of Living Standards 2001 and 2007 - RETA–6515 Country Brief. Manila: ADB.

Ravishankar, N., Gubbins, P., Cooley, R. J., Leach-Kemon, K., Michaud, C. M., Jamison, D. T., & Murray, C. J. 2009. Financing of global health: tracking development assistance for health from 1990 to 2007. The Lancet, 373(9681), 2113-2124.

Selden, T. M. and M. J. Wasylenko. 1992. Benefit incidence analysis in developing countries, No 1015, Policy Research Working Paper Series, The World Bank.

Sridhar, D., and R. Batniji. 2008. "Misfinancing global health: a case for transparency in disbursements and decision making." The Lancet 372, no. 9644: 1185-1191.

The World Bank. 2009. Core Guidance: Preparing Public Expenditure Reviews for Human Development. The World Bank: Washington, DC.

van de Walle, D. 1992. The distribution of the benefits from social services in Indonesia, 1978-87. Policy, Research working papers; no. WPS 871. Public economics. Washington, DC: World Bank. http://documents.worldbank.org/curated/en/1992/03/699739/distribution-benefits-social-services-indonesia-1978-87

van de Walle, D. 2002. Poverty and transfers in Yemen. Middle East and North Africa working paper series; no. 30. Washington, DC: World Bank. http://documents.worldbank.org/curated/en/2002/12/2622228/poverty-transfers-yemen

Wagstaff, A. 2010. Are Government Health Expenditures More Pro-rich Than We Think?, (March).

Wijemanne, N., C. Anuranga, P. Perera, S. Saleem, J. Chandrasiri, A. S. Salehi, K. M. A. Saeed, S. M. Y. Azimi and R. Rannan-Eliya. 2014. The Impact of Out-of-Pocket Expenditure on Poverty and Inequalities in Use of Overall, Maternal and Child Health services in Afghanistan: Evidence from the Afghanistan Health Surveys (2006, 2012) and National Risk and Vulnerability Assessments (2005, 2007). Colombo and Kabul: Institute for Health Policy and Ministry of Public Health Afghanistan.

World Bank. 2010. Promoting the Institutionalization of National Health Accounts: A Global Strategic Action Plan, 2010. http://siteresources.worldbank.org/INTHSD/Resources/376278-1261143298590/GSAPVersion2.0.pdf.

World Bank. 2014a. Health Equity Chapter 8. http://siteresources.worldbank.org/INTPAH/Resources/Publications/459843-1195594469249/HealthEquityCh8.pdf DOA: November 4th, 2014.

World Bank. 2014b. Quantitative Techniques for Health Equity Analysis—Technical Note #6. http://siteresources.worldbank.org/INTPAH/Resources/Publications/Quantitative-Techniques/health_eq_tn06.pdf. DOA: November 4th, 2014.

World Health Organization (WHO). 2001. Macroeconomics and health; Investing in health for economic development. Geneva.
