# Supplementary material for: Countdown to 2015 country case studies: what can analysis of national health financing contribute to understanding MDG 4 and 5 progress?
Source: BMC Public Health. 2016 Sep 12;16(Suppl 2):792. doi: 10.1186/s12889-016-3403-4 (PMC5025819; doi:10.1186/s12889-016-3403-4)
Supplement: Additional file 2: — Country specific health spending data tables. (DOCX 44 kb) [file 12889_2016_3403_MOESM2_ESM.docx]

**Additional file 2**. Country Data Tables

| **Afghanistan Health Expenditure Data** | | |
| --- | --- | --- |
|  | **2008** | **2012** |
| **Total Health Expenditure (THE)** | 1,113,109,636 | 1,500,975,945 |
| External | 203,370,244 | 312,468,367 |
| Govt | 68,133,406 | 84,148,093 |
| Household | 839,322,380 | 1,099,542,464 |
| Other Private | 2,283,607 | 4,817,021 |
| THE as % of GDP | 10.0% | 8.0% |
| % Change in THE |  | 34.85% |
| THE Per Capita | 44.79 | 55.59 |
| % Change of THE Per Capita |  | 24.12% |
| **Reproductive Health Expenditure (RHE)** |  | 246,744,339 |
| External |  | 46,387,936 |
| Govt |  | 6,662,097 |
| Household |  | 193,694,306 |
| Other Private |  | 24,674 |
| RHE as % of THE |  | 16.40% |
| % Change of RHE |  |  |
| RHE Per Capita (women of reproductive age) |  | 44.00 |
| % Change of RHE Per Capita (women of reproductive age) |  |  |
| **Child Health Expenditure (CHE)** |  |  |
| External |  |  |
| Govt |  |  |
| Household |  |  |
| Other Private |  |  |
| CHE as % of THE |  |  |
| % Change of CHE |  |  |
| CHE Per Capita (children under-5 years) |  |  |
| % Change of CHE Per Capita |  |  |

| **Ethiopia Health Expenditure Data** | | | | | |
| --- | --- | --- | --- | --- | --- |
|  | **1995** | **2000** | **2005** | **2008** | **2011** |
| **Total Health Expenditure (THE)** | 346,665,317.20 | 473,991,349.43 | 613,345,295.93 | 1,268,161,518.77 | 1,675,937,431.65 |
| External | 6,309,308.77 | 75,838,615.91 | 226,079,076.08 | 497,626,579.97 | 836,292,778.39 |
| Govt | 134,575,476.14 | 156,417,145.31 | 187,315,653.38 | 282,419,570.23 | 261,446,239.34 |
| Household | 182,553,956.04 | 170,636,885.79 | 188,113,002.26 | 470,361,107.31 | 564,790,914.46 |
| Other Private | 23,261,242.78 | 71,098,702.41 | 11,837,564.21 | 17,881,077.41 | 16,759,374.32 |
| THE as % of GDP | 3.83% | 4.40% | 5% | 4.50% | 5.20% |
| THE Per Capita | 6.16 | 7.47 | 8.39 | 17.16 | 21.20 |
| **Reproductive Health Expenditure (RHE)** |  |  | 75,830,181.89 | 160,954,435.33 | 228,726,052.21 |
| External |  |  | 33,365,280.03 | 57,943,596.72 | 107,501,244.54 |
| Govt |  |  | 14,407,734.56 | 45,067,241.89 | 56,724,060.95 |
| Household |  |  | 28,057,167.30 | 40,238,608.83 | 63,128,390.41 |
| Other Private |  |  |  | 17,704,987.89 | 1,372,356.31 |
| RHE as % of THE |  |  | 12% | 13% | 14% |
| RHE Per Capita (women of reproductive age) |  |  | 4.34 | 8.53 | 12.25 |
| **Child Health Expenditure (CHE)** |  |  | 119,113,685.65 | 121,673,958.00 | 188,335,682.40 |
| External |  |  | 41,689,789.98 | 76,654,593.54 | 51,038,969.93 |
| Govt |  |  | 27,396,147.70 | 14,600,874.96 | 46,707,249.23 |
| Household |  |  | 50,027,747.97 | 29,201,749.92 | 90,212,791.87 |
| Other Private |  |  |  | 1,216,739.58 | 376,671.36 |
| CHE as % of THE |  |  | 19% | 10% | 11% |
| CHE Per Capita (children under-5 years) |  |  | 9.24 | 9.38 | 16.33 |

| **Malawi Health Expenditure Data** | | | | | | | | | | |
| --- | --- | --- | --- | --- | --- | --- | --- | --- | --- | --- |
|  | **2003** | **2004** | **2005** | **2006** | **2007** | **2008** | **2009** | **2010** | **2011** | **2012** |
| **Total Health Expenditure (THE)** | 134,097,821 | 188,486,715 | 219,300,626 | 351,090,568 | 408,111,018 | 489,703,351 | 548,564,851 | 556,585,118 | 643,942,971 | 637,767,573 |
| External | 61,523,580 | 122,127,775 | 134,804,857 | 246,385,779 | 284,874,077 | 303,160,117 | 359,444,977 | 346,195,943 | 412,767,444 | 450,263,907 |
| Govt | 47,441,260 | 39,702,953 | 53,685,602 | 75,364,350 | 55,082,401 | 109,659,807 | 98,606,674 | 125,788,237 | 141,667,454 | 102,680,579 |
| Household | 16,401,941 | 18,337,624 | 20,063,498 | 32,240,455 | 48,973,322 | 52,398,259 | 63,633,523 | 62,894,118 | 60,530,639 | 66,965,595 |
| Other Private | 8,710,208 | 8,318,363 | 10,746,669 | 31,584,317 | 19,181,218 | 24,485,168 | 26,879,678 | 21,706,820 | 28,977,434 | 17,857,492 |
| THE as % of GDP | 9.9% | 12.6% | 12.8% | 9.8% | 11.2% | 12.2% | 11.6% | 7.4% | 8.2% | 9.0% |
| THE Per Capita | 18.72 | 20.66 | 23.51 | 28.47 | 31.45 | 37.22 | 41.20 | 36.43 | 40.42 | 39.3 |
| **Reproductive Health Expenditure (RHE)** | 39,354,150 | 38,315,246 | 38,381,718 |  | 33,389,721 | 42,494,765 | 50,761,343 | 52,770,237 | 75,822,135 | 63,573,728 |
| External | 15,348,118 | 21,439,765 | 19,512,809 |  | 24,708,393 | 29,321,388 | 37,309,587 | 33,020,083 | 49,216,039 | 44,134,112 |
| Govt | 16,335,489 | 8,700,307 | 11,130,698 |  | 4,774,730 | 8,881,406 | 8,223,338 | 13,817,411 | 19,606,480 | 14,139,834 |
| Household | 5,102,209 | 6,108,297 | 5,326,130 |  | 3,172,023 | 3,357,086 | 4,111,669 | 3,218,984 | 3,218,992 | 3,229,633 |
| Other Private | 2,683,564 | 2,066,877 | 2,602,913 |  | 734,574 | 934,885 | 1,167,511 | 2,660,909 | 3,780,624 | 2,070,149 |
| RHE as % of THE | 19% | 16% | 14% |  | 8.20% | 8.70% | 9.30% | 8% | 12% | 14% |
| Estimated RHE Per Capita (women of reproductive age) | 14.26 | 13.46 | 13.06 |  | 10.67 | 13.17 | 15.28 | 15.44 | 21.59 | 17.63 |
| **Child Health Expenditure (CHE)** | 34,622,203 | 32,026,797 | 42,147,216 |  | 28,298,262 | 42,440,443 | 46,644,677 | 80,719,268 | 89,450,175 | 115,076,974 |
| External | 13,502,659 | 15,693,130 | 20,652,136 |  | 12,168,253 | 21,771,947 | 22,576,024 | 48,410,397 | 50,184,372 | 83,807,294 |
| Govt | 14,195,103 | 8,967,503 | 12,644,165 |  | 4,606,575 | 8,144,524 | 8,807,279 | 18,275,595 | 25,084,723 | 17,596,043 |
| Household | 5,193,330 | 5,764,823 | 6,322,082 |  | 10,470,357 | 11,161,836 | 13,526,956 | 11,381,119 | 10,822,234 | 11,479,497 |
| Other Private | 1,731,110 | 1,601,340 | 2,528,833 |  | 1,075,334 | 1,358,094 | 1,725,853 | 2,652,156 | 3,358,845 | 2,194,140 |
| CHE as % of THE | 17% | 14% | 16% |  | 6.90% | 8.70% | 8.60% | 15% | 14% | 18% |
| CHE Per Capita (children under-5 years) | 14.97 | 13.37 | 17.63 |  | 12.83 | 18.41 | 19.38 | 32.64 | 34.91 | 44.00 |

| **Tanzania Health Expenditure Data** | | | |
| --- | --- | --- | --- |
|  | **2003** | **2006** | **2010** |
| **Total Health Expenditure (THE)** | 930,175,331 | 1,619,285,383 | 1,735,529,197 |
| External | 255,239,521 | 712,485,714 | 686,883,227 |
| Govt | 236,543,441 | 453,399,835 | 451,208,438 |
| Household | 390,952,224 | 404,821,573 | 560,570,386 |
| Other Private | 47,438,944 | 48,578,261 | 36,867,146 |
| THE as % of GDP | 5.0% | 7.6% | 8.2% |
| THE Per Capita | 26.20 | 43.28 | 43.17 |
| **Reproductive Health Expenditure (RHE)** | 124,779,000 | 176,523,300 | 329,560,830 |
| External | 26,952,264 | 38,129,033 | 100,252,404 |
| Govt | 43,048,755 | 78,023,299 | 69,932,808 |
| Household | 50,161,158 | 52,427,420 | 155,980,130 |
| Other Private | 4,616,823 | 7,943,549 | 3,394,477 |
| RHE as % of THE | 13.70% | 10.70% | 17.90% |
| RHE Per Capita (women of reproductive age) |  |  |  |
| **Child Health Expenditure (CHE)** |  |  | 173,519,568 |
| External |  |  | 23,251,622 |
| Govt |  |  | 47,891,401 |
| Household |  |  | 97,865,036 |
| Other Private |  |  | 4,511,509 |
| CHE as % of THE |  |  | 9.40% |
| CHE Per Capita (children under-5 years) |  |  | 23.16 |

| **Peru Health Expenditure Data** | | | | | | | | | | | | | |  |
| --- | --- | --- | --- | --- | --- | --- | --- | --- | --- | --- | --- | --- | --- | --- |
|  | **2000** | **2001** | **2002** | **2003** | **2004** | **2005** | **2006** | **2007** | **2008** | **2009** | **2010** | **2011** | **2012** | **2013** |
| **Total Health Expenditure (THE)** | 3,590,855,785 |  | 3,853,260,104 | 3,918,352,460 | 4,081,043,009 | 4,087,831,760 | 4,902,441,002 | 5,699,182,956 | 6,599,766,319 | 7,354,459,877 | 8,430,100,319 | 9,238,688,016 | 10,851,132,614 |  |
| External | 39,499,414 |  | 34,679,341 | 39,183,525 | 40,810,430 | 40,878,318 | 53,926,851 | 68,390,195 | 72,597,430 | 66,190,139 | 67,440,803 | 64,670,816 | 97,660,194 |  |
| Govt | 847,441,965 |  | 1,032,673,708 | 1,061,873,517 | 1,159,016,215 | 1,083,275,416 | 1,152,073,635 | 1,327,909,629 | 1,478,347,655 | 2,029,830,926 | 2,377,288,290 | 2,503,684,452 | 3,146,828,458 |  |
| Household | 1,411,206,324 |  | 1,525,891,001 | 1,512,484,050 | 1,505,904,870 | 1,541,112,573 | 1,990,391,047 | 2,365,160,927 | 2,804,900,685 | 2,912,366,111 | 3,279,309,024 | 3,584,610,950 | 4,014,919,067 |  |
| Other Private | 25,135,990 |  | 26,972,821 | 27,428,467 | 28,567,301 | 28,614,822 | 29,414,646 | 39,894,281 | 46,198,364 | 44,126,759 | 50,580,602 | 267,921,952 | 271,278,315 |  |
| Social Insurance  (HH+Govt) | 1,271,162,948 |  | 1,236,896,493 | 1,281,301,255 | 1,350,825,236 | 1,389,862,798 | 1,671,732,382 | 1,897,827,924 | 2,191,122,418 | 2,309,300,402 | 2,655,481,600 | 2,808,561,157 | 3,320,446,580 |  |
| THE as % of GDP | 4.85% |  | 4.98% | 4.69% | 4.63% | 4.77% | 4.44% | 4.58% | 4.74% | 5.36% | 5.03% | 4.93% | 4.56% |  |
| % Change in THE |  |  |  |  |  |  |  |  |  |  |  |  |  |  |
| THE Per Capita | 94.72 |  | 100.58 | 100.18 | 110.82 | 127.18 | 137.99 | 164.33 | 199.72 | 223.10 | 253.71 | 282.10 | 331.00 |  |
| % Change of THE Per Capita |  |  |  | -0.39% | 10.62% | 14.76% | 8.50% | 19.08% | 21.53% | 11.71% | 13.72% | 11.19% | 17.34% |  |
| **Reproductive Health Expenditure (RHE) (maternal and neonatal)** |  |  |  | 98,773,865 | 93,892,610 | 126,522,897 | 120,818,105 | 120,839,780 | 129,599,764 | 168,680,092 | 187,246,481 | 239,853,100 | 379,941,297 |  |
| External |  |  |  | 8,870,000 | 3,850,000 | 9,430,000 | 7,680,000 | 7,870,000 | 6,890,000 | 12,580,000 | 12,290,000 | 6,920,000 | 5,930,000 |  |
| Govt | 26,380,000 |  | 22,450,000 | 133,190,000 | 123,380,000 | 157,710,000 | 138,840,000 | 134,530,000 | 143,150,000 | 179,390,000 | 188,760,000 | 237,940,000 | 374,010,000 |  |
| Household |  |  |  |  |  |  |  |  |  |  |  |  |  |  |
| Other Private |  |  |  |  |  |  |  |  |  |  |  |  |  |  |
| RHE as % of THE | 0.73% |  | 0.58% | 3.64% | 3.14% | 3.67% | 3.08% | 2.57% | 2.26% | 2.61% | 2.53% | 2.95% | 3.92% |  |
| % Change of RHE |  |  |  | 6.31% | 0.86% | 0.17% | 0.84% | 0.84% | 1.16% | 0.97% | 1.17% | 1.33% | 1.02% |  |
| RHE Per Capita (women of reproductive age) | 30.93 | 34.48 | 25.91 | 152.11 | 142.57 | 182.69 | 191.33 | 187.25 | 192.80 | 243.36 | 244.70 | 323.35 | 512.10 | 574.00 |
| % Change of RHE Per Capita (women of reproductive age) |  | 11% | -25% | 487% | -6% | 28% | 5% | -2% | 3% | 26% | 1% | 32% | 58% | 12% |
| **Child Health Expenditure (CHE)** |  |  |  | 26,093,828 | 69,052,208 | 75,044,790 | 85,016,932 | 128,306,938 | 398,813,265 | 217,920,737 | 297,039,466 | 302,187,149 | 442,483,671 |  |
| External |  |  |  | 5,890,000 | 11,800,000 | 21,280,000 | 18,870,000 | 22,740,000 | 22,300,000 | 58,830,000 | 22,660,000 | 9,960,000 | 7,920,000 |  |
| Govt | 24,980,000 |  | 29,880,000 | 31,640,000 | 81,770,000 | 77,860,000 | 84,220,000 | 128,460,000 | 439,420,000 | 189,190,000 | 296,280,000 | 298,540,000 | 434,560,000 |  |
| Household |  |  |  |  |  |  |  |  |  |  |  |  |  |  |
| Other Private |  |  |  |  |  |  |  |  |  |  |  |  |  |  |
| CHE as % of THE | 1.10% |  | 0.90% | 0.80% | 1% | 2.30% | 2.20% | 2.70% | 7% | 3.40% | 4% | 3.70% | 4.60% |  |
| % Change of CHE |  |  |  | 1.25% | 2.40% | 0.94% | 0.10% | 1.26% | 2.54% | 0.49% | 1.19% | 9.27% | 1.23% |  |
| CHE Per Capita (children under-5 years) | 7.47 | 9.59 | 8.81 | 9.23 | 24.55 | 23.28 | 26.54 | 40.53 | 136.18 | 59.93 | 98.34 | 101.46 | 148.60 | 175.90 |
| % Change of CHE Per Capita |  | 28% | -8% | 5% | 166% | -5% | 14% | 53% | 236% | -56% | 64% | 3% | 46% | 18% |
|  |  |  |  |  |  |  |  |  |  |  |  |  |  |  |

| **Pakistan Health Expenditure Data** | | | | |
| --- | --- | --- | --- | --- |
|  | **2006** | **2008** | **2010** | **2012** |
| **Total Health Expenditure (THE)** | 5,350,841,736 | 5,250,928,484 | 5,541,810,698 | 5,927,756,635 |
| External | 67,362,634 | 66,459,397 | 63,006,160 | 102,414,260 |
| Govt | 1,226,963,618 | 1,352,148,844 | 1,753,321,268 | 2,190,883,537 |
| Household | 3,642,249,454 | 3,442,863,330 | 3,358,643,564 | 3,250,927,339 |
| Other Private | 414,266,029 | 389,456,913 | 366,839,707 | 383,531,499 |
| THE as % of GDP | 2.59% | 3.36% | 3.00% | 2.80% |
| % Change in THE |  | -1.87% | 5.54% | 6.96% |
| THE Per Capita |  | 37.32 | 32.85 | 34.70 |
| % Change of THE Per Capita |  |  | -11.98% | 5.63% |
| **Reproductive Health Expenditure (RHE)** |  |  |  |  |
| External |  |  |  |  |
| Govt |  |  |  |  |
| Household |  |  |  |  |
| Other Private |  |  |  |  |
| RHE as % of THE |  |  |  |  |
| % Change of RHE |  |  |  |  |
| RHE Per Capita (women of reproductive age) |  |  |  |  |
| % Change of RHE Per Capita (women of reproductive age) |  |  |  |  |
| **Child Health Expenditure (CHE)** |  |  |  |  |
| External |  |  |  |  |
| Govt |  |  |  |  |
| Household |  |  |  |  |
| Other Private |  |  |  |  |
| CHE as % of THE |  |  |  |  |
| % Change of CHE |  |  |  |  |
| CHE Per Capita (children under-5 years) |  |  |  |  |
| % Change of CHE Per Capita |  |  |  |  |
