# Supplementary material for: Countdown to 2015 country case studies: what can analysis of national health financing contribute to understanding MDG 4 and 5 progress?
Source: BMC Public Health. 2016 Sep 12;16(Suppl 2):792. doi: 10.1186/s12889-016-3403-4 (PMC5025819; doi:10.1186/s12889-016-3403-4)
Supplement: Additional file 3: — Health spending data for all Countdown countries by income status in 2010. (DOCX 219 kb) [file 12889_2016_3403_MOESM3_ESM.docx]

**Additional file 3**. Health spending data for all Countdown countries by income status in 2010


**Per Capita Health Expenditure (constant 2012 US$)^a^**

**Total Health Expenditure as a Percent of GDP^a^**

^a^ In the graphs above, the “other” Countdown countries (those not included in this study) are illustrated in blue nodes, while the Countdown countries included in this study have red nodes and labelled. Both Ethiopia and Afghanistan 2010 THE estimates as a percent of GDP were estimated using linear interpolation.
