# Supplementary material for: Countdown to 2015 country case studies: what can analysis of national health financing contribute to understanding MDG 4 and 5 progress?
Source: BMC Public Health. 2016 Sep 12;16(Suppl 2):792. doi: 10.1186/s12889-016-3403-4 (PMC5025819; doi:10.1186/s12889-016-3403-4)
Supplement: Additional file 4: — GDP per capita trend data by country. (DOCX 707 kb) [file 12889_2016_3403_MOESM4_ESM.docx]

**Additional file 4**. GDP per capita trend data by country
